# PDMS Microfabrication and Design for Microfluidics and Sustainable Energy Application: Review

**DOI:** 10.3390/mi12111350

**Published:** 2021-10-31

**Authors:** Lin Lin, Chen-Kuei Chung

**Affiliations:** Department of Mechanical Engineering and Core Facility Center, National Cheng Kung University, Tainan 701, Taiwan; N16091621@gs.ncku.edu.tw

**Keywords:** polydimethylsiloxane, PDMS, microfluidics, micromixer, micropump, capillary, biomedical, triboelectrical nanogenerator, TENG, sustainable energy

## Abstract

The polydimethylsiloxane (PDMS) is popular for wide application in various fields of microfluidics, microneedles, biology, medicine, chemistry, optics, electronics, architecture, and emerging sustainable energy due to the intrinsic non-toxic, transparent, flexible, stretchable, biocompatible, hydrophobic, insulating, and negative triboelectric properties that meet different requirements. For example, the flexibility, biocompatibility, non-toxicity, good stability, and high transparency make PDMS a good candidate for the material selection of microfluidics, microneedles, biomedical, and chemistry microchips as well as for optical examination and wearable electronics. However, the hydrophobic surface and post-surface-treatment hydrophobic recovery impede the development of self-driven capillary microchips. How to develop a long-term hydrophilicity treatment for PDMS is crucial for capillary-driven microfluidics-based application. The dual-tone PDMS-to-PDMS casting for concave-and-convex microstructure without stiction is important for simplifying the process integration. The emerging triboelectric nanogenerator (TENG) uses the transparent flexible PDMS as the high negative triboelectric material to make friction with metals or other positive-triboelectric material for harvesting sustainably mechanical energy. The morphology of PDMS is related to TENG performance. This review will address the above issues in terms of PDMS microfabrication and design for the efficient micromixer, microreactor, capillary pump, microneedles, and TENG for more practical applications in the future.

## 1. Introduction

Polydimethylsiloxane (PDMS) is a well-known functional material in fields of architecture [1,2], optical performance enhancement [3,4,5,6,7], microfluidics [8,9,10,11,12,13,14,15], chemistry and biomedical [16,17], microneedles [18,19,20,21,22,23,24,25], and the sustainable energy application of a triboelectrical nanogenerator (TENG) [26,27,28,29,30,31,32,33]. Solid PDMS has advantages of non-toxic, hydrophobic, transparent, and controllable Young’s modulus. The material cost of PDMS is much lower than that of silicon and glass wafers, and its light transmittance, good biocompatibility, and its ability to easily bond with a variety of materials at room temperature attract much attention. Several polymer materials such as polymethyl methacrylate (PMMA), PDMS, polycarbonate, cycloolefin polymer, and copolymers, and so on have also been used for microsystem technology combined with the Si and glass micromachining, bonding, and/or surface modification [34]. In terms of the microfabrication and design of microfluidics application, the micropumps, micromixers, and/or microreactors are the basic components. The active micromixers and micropumps are involved in the high-cost complicated fabrication and external power input, therefore the passive micromixers and self-driven capillary pumping are more popular and widely studied [34]. Passive micromixers with effective mixing efficiency, the capillary pumping with long-term hydrophilicity, and an easy fabrication process are the main goal for research and practical use. The early microfluidics and biomedical template are based on the silicon or glass substrate combined with the photolithography process by SU-8 thick photoresist; some laser ablated polymer PMMA templates are also applied due to low cost and easy fabrication [34,35,36,37]. The above SU-8 and PMMA templates for casting PDMS microchannels are widely used in microfluidics. The PDMS microfluidics offer several advantages such as flexibility, low environmental impact, biocompatibility, and good chemical stability for bulk production, making them the most promising materials for microsystem technology for micromixers and micropumps. In terms of microfabrication and design of PDMS advances for microneedles [18,19,20,21,22,23,24,25] and for TENG sustainable energy application, the laser ablated PMMA master mold is used to fabricate various PDMS microstructures. The TENG fabrication is fast and cost effective for harvesting mechanical energy. Selecting two suitable triboelectric materials can produce high open-circuit voltage and enough power for portable electronic devices of which the TENG performance is related to the material microstructure, process parameters, and device assembly. This sustainable energy application has greatly been developed in wearable devices, sensors, and human-machine interfaces from now to the future [30,31,32,33,38,39].

In this review, we look forward to introducing PDMS properties, microfabrication, and design for microfluidics and recent sustainable energy application. Besides highlighting the traditional microfabrication of Si and glass materials using the photolithography, etching and deposition processes, we pay great attention to the laser micromachining on polymer materials of PMMA and PDMS to produce the desired microstructure and the master mold that is followed by the one-step and two-step casting method for duplicating the dual-tone convex and concave structures for different applications according to the designed dimension and specification of devices. The laser micromachining merits a simple, fast, and green process but encounters some ablation problems including bulges, resolidification, and scorches. The key issues of process improvement on the laser ablation problems are addressed using photoresist polymer coating and a metal foil-assisted laser ablation. The hydrophobic recovery of PDMS microchannel after oxygen plasma treatment is a big problem for long-term hydrophilic microfluidic mixers and a capillary pump. The effective method of using polyethylene glycol (PEG) coating to overcome the hydrophobic recovery problem is raised for a long-term self-driven capillary micromixer and micropumps. Extending the PEG coating characteristics, the dual-tone PDMS-to-PDMS microstructures can be made without the stiction problem using the novel two-step casting from the laser ablated PMMA master mold for the microfluidic application. Recently, PDMS microneedles arrays formed from the laser ablated PMMA deep concave mold are also used for the TENG as the mechanical energy harvester and will be discussed in detail.

### 1.1. PDMS Properties

**Figure 1:**
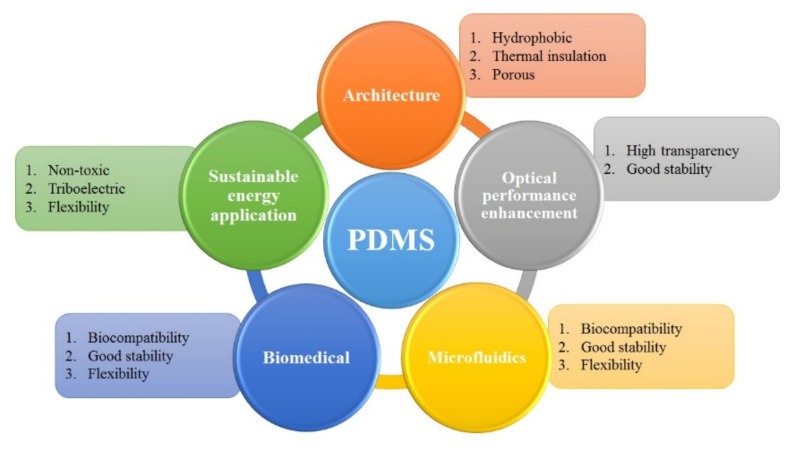
The PDMS applications corresponding to its properties.

The characteristics of PDMS make it become a well-known material, the applications corresponding to each feature is shown in Figure 1. Its excellent light transmittance is often used in optical research and anti-reflective film applications. By mixing elastomer and curing agent to adjust the Young’s coefficient and thermosetting characteristic, the required micro-nano structure can be achieved and adjusted to meet the applications. The non-toxic, biocompatible, good stability, and flexible properties have made PDMS a well-known microfluidics template, which will be discussed in this short review. With integrating the laser processing, the advantages of reducing the cost and shortening the process time can be achieved. Furthermore, researchers have also focused on the triboelectric/dielectric properties, including a variety of sensors [40,41,42,43,44,45,46,47,48,49] produced by composite structures from PDMS and other materials. Triboelectric properties are the main reason for research on TENG in recent years. Compared with other triboelectric materials, PDMS has the advantages of good flexibility, being light weight, and having good biocompatibility. In addition to power generation, it is currently used in sensing, wearable devices, and human machine interface applications.

### 1.2. Microfabrication and Surface Modification for PDMS Microfluidics

Materials such as Si, glass, and polymer materials have been widely used for the micro(/nano)-electro-mechanical system (MEMS/NEMS). The traditional microfabrication uses photolithography and etching for patterning Si and glass [50,51,52,53,54,55,56,57,58,59,60]. For example, the anisotropic wet etching of Si is often performed by potassium hydroxide (KOH) and tetramethylammonium hydroxide (TMAH) while the isotropic wet etching of Si is done by a hydrofluoric acid (HF) and nitric acid (HNO_3_) based solution. The dry etching of Si is also conducted by reactive ion etching (RIE) and/or deep reactive ion etching (DRIE) for transferring the photolithography resist pattern into silicon for a vertical profile. Several drawbacks, such as high cost, long process times, and a limited machining profile exist and need modifying process in different applications. Regarding the glass chip fabrication, it also employs the photolithography and dry/wet etching processing [50,51,57,58,59]. Iliescu et al. [51] proposed and compared three etching masks of Cr/Au, Cr/Cu, and PECVD amorphous silicon developed for Pyrex glass micromachining in hydrofluoric acid solution. Baram et al. [59] used RIE and standard lithography processes for Pyrex glass at a high etch rate to create deep cavities. Tang et al. [61] proposed a facile method with a hydrophobic layer followed by hydrophilic resist photolithography for an etching-free process to produce a binary wettability patterned microstructure for microfluidics. Overall, the fabrication of Si and glass microchips is time consuming, costly, and/or has high-temperature fabrication. It is noted that the microfluidic chips have attracted much attention to fluid mixing, pumping, and control for the lab-on-a-chip application recently [62,63,64]. The polymer microfluidic chips benefit low-cost, easy fabrication, and biocompatibility for the great research interests in rapid prototyping, biochips, portable, and wearable devices. The high-hydrophilic surface of channels is a crucial issue for self-driven microfluidic systems to effectively actuate liquid without any additional pump. To acquire a better hydrophilicity performance, the surface modification is usually applied to change the inherent hydrophobic property of PDMS or SU-8 microfluidic channels [65,66,67,68,69,70,71]. However, there is hydrophobic recovery problem of the PDMS surface with a time-limited hydrophilicity modified using traditional plasma. Therefore, to obtain a long-term microfluidic chip, the inherent hydrophilic glass is a good replacement, but needs to overcome more complicated and time-consuming processing [72,73].

For a rapid fabrication of microfluidic chips, the laser micromachining is a good candidate due to the advantages of having a simple, fast, and direct-write process for different geometrical shapes compared with a traditional photolithography and etching method. Therefore, it is feasible to use CO_2_ laser ablation and/or polymer molding to fabricate a PMMA or PDMS fluidic microstructure for a micromixer, capillary pumping, and bio-MEMS applications. A common integration process using laser ablated PMMA mold, followed by PDMS casting and surface modification can be fabricated for a wide microstructure and various applications. In recent years, the PDMS-based microfluidic system is widely investigated [74,75,76,77,78,79,80,81,82,83,84,85,86]. The hydrophobic property of PDMS is another issue to be improved for enhancing mixing efficiency. In addition, how to fabricate the dual-tone convex-and-concave PDMS microstructure for microfluidic chip is another challenge. The surface modification using hydrophilic PEG material is important for hydrophilicity enhancement of PDMS for the self-driven microfluidic chip as well as for the 2-step PDMS casting for a dual-tone microstructure. Lai & Chung [87,88] proposed that the characteristic flow and mixing behavior, surface hydrophilicity, and optic property of the PEG-coated PDMS microfluidic chip were deeply investigated to show the merits of long-term self-driven microfluidic chips. It is noted that for PDMS casting in different-dimension features, both the laser machined PMMA and photolithography SU-8 on silicon are usually selected as the desired molds. They both have their own benefits and drawbacks. The PMMA mold, ablated by a CO_2_ laser, is cheap and simple but has a rough surface and larger processing dimension, generally larger than several hundreds of microns. On the contrary, the silicon mold, which applied the patterned SU-8 photoresist film with specific morphology is costly and complicated but has a smooth surface and a precise dimension from tens to hundreds of microns. In chapter 4, we will discuss in detail these two kinds of molds and their application of microfluidic chips.

### 1.3. Design of Polymer Microstructure for Microfluidics

High-efficiency mixing in a micromixer is crucial for reaction and detection uniformity in microfluidic systems [89,90,91,92,93,94,95,96,97,98,99], a micro total analysis system (µ-TAS), or lab-on-a-chip (LOC) applications [100,101,102,103,104,105]. The micromixers are divided into two main kinds of types i.e., active and passive ones to study the design, structure, fabrication, mechanism, and assembly on mixing enhancement. Lukyanenko et al. [89] proposed that an active mixer for dried reagents was made from an electro-mechanical speaker’s membrane which was connected to the input of the chip. Cortelezzi et al. [90] presented a geometrically scalable active micro-mixer suitable for biomedical and bioengineering applications and potentially assimilable in a lab-on-chip. Compared to the passive ones, the active microfluidic system may bring benefit to good mixing efficiency at a shorter channel length with less mixing time using the acoustic force and magneto hydrodynamics mixers. However, there are accompanying problems such as the extra power source, complex structure, time lengths, and high-cost fabrication. On the other hand, the passive microfluidic system has the advantages of concise design and easy fabrication for good mixing under proper design of the microstructure. The passive microfluidic system generally has two types of microchannel structures i.e., the 3D structure and the 2D planar one to mix fluids through the convection-chaotic-vortex effect or the diffusion effect. Bell et al. [91] proposed a planar gravity perfusion system that exploits the strong capillary action in the flow chamber as a passive limit-valve. Gidde et al. [92] presented that passive planar micromixers based on circular and square mixing chambers spaced at an equidistant along the length of a micromixer could operate in the laminar flow regime for a high mixing index. Rampalli et al. [93] proposed that a serpentine square convergent-divergent micromixer is a simple and effective passive device for micromixing. Compared to 2D planar passive microfluidics, the 3D ones [106,107,108,109,110,111,112] benefit the structure-enhanced repeated mixing for good efficiency.

The PDMS is a popular material for microfluidics. A layer-by-layer process could make a 3D microfluidic mold for casting PDMS. The 2D PDMS layers may be combined together to compose 3D structures. However, 3D micromixers have drawbacks such as the complex fabrication process and being time consuming, which are difficult to commercialize. In contrast, the planar micromixers can be fabricated in a simple process but demand a long mixing channel [113] or high pressure drop [8] for enhancing mixing. When the straight microchannel is used for good diffusion mixing, it needs the longer microchannel e.g., a 15–20-mm mixing channel length and much time. So, Ma et al. [114] proposed that a microchannel with an active micropump can reduce the mixing time in a much shorter channel length with the help of an extra power source or fluid vibration. However, the included active micropump makes the design and fabrication more complicated together with high cost and a long process time. In such an issue, the 2D passive micromixer with the proper microchannel design can greatly enhance the mixing efficiency at a short mixing length [8,9,10]. In-between the mentioned active and passive microfluidics, the self-driven microfluidics which is actuated by a capillary force without an extra power supply attract wide research [115,116,117,118]. However, low transport velocity and hydrophobic recovery of the surface-treated material are the main drawbacks. The surface treatment of PDMS for a low contact angle of hydrophilic property is widely studied. One key issue is how to conquer the short-term hydrophobic recovery of PDMS that hinders the development of the self-driven capillary microchips. The PEG coated PDMS microfluidic chips are feasible for a long-term capillary-driven microfluidic application with the high-wetting property for rapid fluid transport [87,88]. The capillary-driven micromixer has great potential for bio-MEMS applications because of low pressure drop, rapid mixing, and low cost. In this review, the PDMS based 2D passive micromixer with the baffled and rhombic microchannel design is addressed for mixing efficiency enhancement. In brief, Dean Vortices and the recirculation effect are two major mixing effects. The rhombic microchannel may induce the Dean Vortices effect at higher Reynolds (*Re*) number. Beside the continuous-flow system, the microreactor is discussed to synthesize nanoparticles materials because of some advantages, such as high surface area to volume ratio and ultra-fast mixing. However, a rising problem of the nanoparticle synthesis in the microreactor is the low production rate due to the small radius of the microchannel and low flow velocity in the microchannel. An obstacled efficient micromixer was combined as high-throughput synthesis system for overcoming the disadvantage mentioned above. The channel design, surface properties of the micromixer, and the flow simulation will be discussed in depth in chapter 4.

### 1.4. Design of PDMS Microstructure for the Sustainable Energy Application

Waste energy harvesting becomes one of the positive approaches in seeking new energy sources from our ambient environment. A considerable amount of research on harvesting lost energy are reported to serve in human daily living like wind, solar, thermal, mechanical, and chemical energy. The new triboelectric nanogenerator (TENG) technology [119] is successfully used for harvesting wasted energies from motion [120], sliding [121,122], vibration [123,124], hydraulic [125], or air power [126,127,128,129,130] and has received much attention for effective harvesting energy in tremendous practical applications including, consumer electronics [131,132,133], biosensors [134,135,136], pressure sensors [137,138,139], humidity or gas sensor [38,140,141,142], and portable electronic devices [39,143,144]. It is important and a big issue for TENG devices to develop a cost-effective, rapid processing, environmentally-friendly, and high-performance technology. Several important factors for promoting TENG are considered including structures, materials, morphology, assembly, and package of the TENG with different types of operation mechanisms. The TENGs are applied as the mechanical wasted energy harvester in harvesting airflow [128,129,130,145], water flow [146,147,148], human-machine-interface smart equipment [149], and human motion [150,151,152].

Most conventional TENGs are based on the friction operation of cycling contact-separate mechanism of two major triboelectric materials. Table 1 lists the positive-to-negative triboelectricity trend of common materials in TENGs [27]. The TENG performance is related to the triboelectricity induced by two triboelectric materials and surfaces with micro/nano structures that is green energy and is greatly used for harvesting the self-power, soft-robot [153], walking energy [154], and large-amplitude spring steel motion energy [155] as well as different directions of wasted energy from the environment. As mentioned, the surface structure and morphology of TENGs, e.g., the cubes, nanorods array [156,157], pyramids [158,159], textiles [160], nano-patterns [161,162,163], and wrinkle [164] are significantly affecting the TENG out performance. Table 2 lists the performance comparison from different surface structures and morphologies. Some limitations exist in the structure, morphology, and process with long-time, high-cost, complex fabrication based on the electrochemical deposition [156], lithography method [159,165,166,167], and/or etching process [159,163]. A facile method using the integrated laser ablation and polymer casting processes mentioned above is practical for producing high contact surface area of the microneedle (MN)-PDMS with Al for high-performance Al/PDMS MN-TENG and application to the self-powered devices and pressure sensor [30,32,33]. The detailed design of MN-PDMS for Al/PDMS TENG is described in chapter 5.

## 2. Laser Ablation and Polymer Casting for Microfluidics, Microneedles, and Sustainable Energy

Laser micromachining or ablation technique has great potential in microfluidic and biomedical, chip, optical/optoelectric devices, miniaturization fabrication, laser selective treatment, and emerging TENG sustainable energy. However, the thermal effect is an important issue during laser processing for surface quality control. Chung et al. [168,169] have proposed an advanced metal-foil assisted laser processing for polymer material or thin film treatment to greatly diminish the thermal defects during micromachining. In the following, they designed some possibility application using the advanced laser processing for the dimension of universal microchannel usually around several hundred micrometers. Compared to the expensive time-consuming photolithography method in early microfluidic chip fabrication, the advanced laser processing on PMMA that is a popular polymer in the microfluidic structures and optical devices offers a facile approach for good quality microfluidic chip fabrication. The simple effective approach was developed for the fabrication of PMMA by reducing the feature size and minimizing bulges, re-solidification, and clogging phenomena from the thermal-induced defects. In addition, the drawbacks of traditional direct laser-machined PDMS are the defects of scorches and re-solidification [168,169]. These results are concerned with the photo-thermal mechanism during laser ablation. The PMMA ablation combined with the PDMS casting is proposed to improve the drawback of direct PDMS ablation in air. Some examples are shown below.

**Figure 2:**
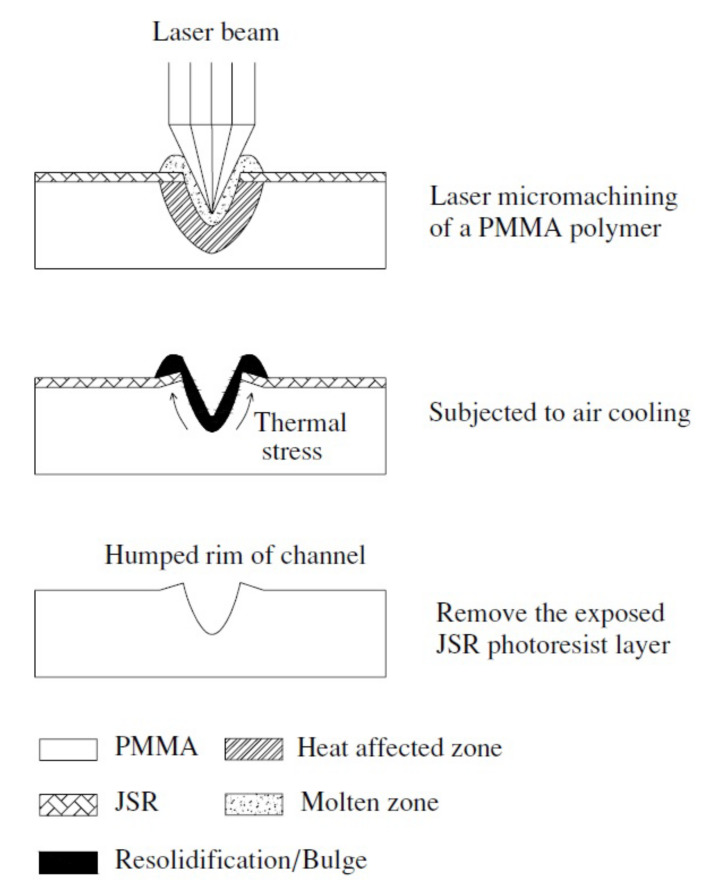
The schematic model of the bulge/hump formation mechanism in the laser machined PMMA with an exposed JSR photoresist cover layer.

The schematic bulge/hump formation mechanism on the rims of a channel in the laser ablated PMMA with an exposed JSR photoresist cover layer is shown in Figure 2 [168]. Two mechanisms are stated for the formation of bulges on the channel rims. The first is due to the thermal molten polymer resolidified by atmospheric air cooling, called the ‘conventional bulge’, which can be removed by the cover layer like photoresist. The second is attributed to the thermal stress or residual stress induced distortion in a large temperature gradient during the laser processing, called a ‘hump’. The thermal stress is generated from the great temperature difference between the thermal unaffected zone, thermal-affected zone (HAZ), molten liquid, and the cover layer. The higher laser ablation passes results in the more heat-induced thermal stress for hump formation. Furthermore, the overall resolidification and thermal-stress induced defects can be significantly diminished by the advanced metal-foil assisted laser processing [169].

**Figure 3:**
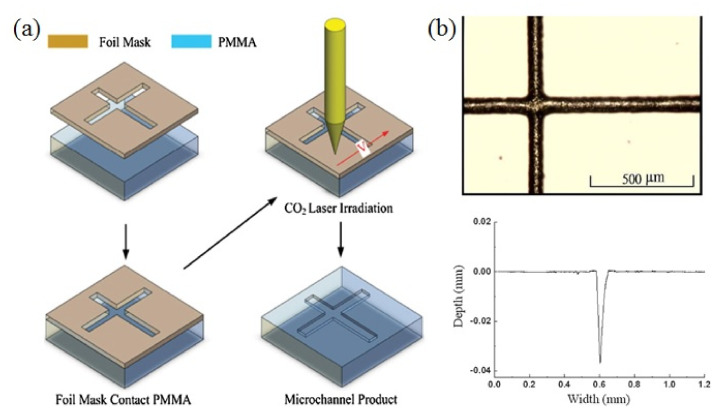
(**a**) Schematic process flow of the FACLAM technique including the metal foil mask on 2 mm PMMA and the scanning CO_2_ laser source. (**b**) Optical micrograph and α-step profile of the PMMA cross-microchannel formed by one-pass ablation.

The schematic process flow of the Foil-Assisted CO_2_ Laser Micromachining (FACLAM) is shown in Figure 3a for ablating the PMMA cross-microchannel. The bulges and feature sizes of channels (Figure 3b,c) can be highly decreased due to reducing the surface temperature with the assistance of a metal mask that may block and conduct the remaining heat out during ablation. It includes the metal foil mask on 2 mm thick PMMA and the scanning CO_2_ laser source. The metal mask made of SUS-304 stainless steel film may physically limit the featured width of channel less than the laser spot size, hinder the bulge formation from resolidification, and reduce the HAZ around the channel by thermally conducting heat away from the ablated channel. It is noted that no clogging is found on the cross junction.

**Figure 4:**
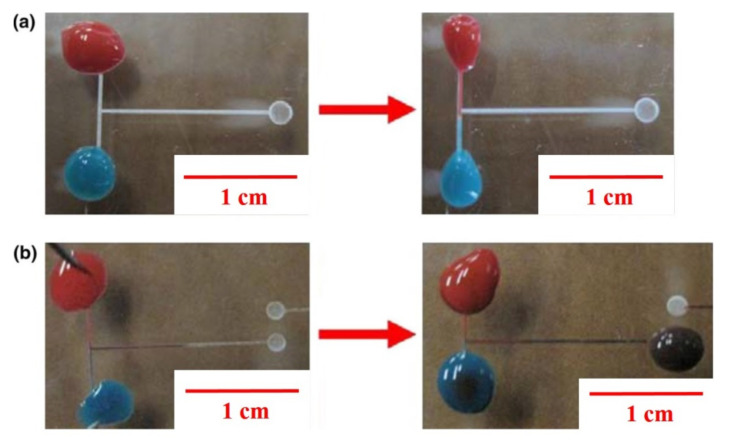
T-channel of PMMA microfluidic device using pigment to dye mixing test: (**a**) Directly in air, (**b**) assisted with metal film.

The red and blue pigment solutions are used to test the mixing behavior. The two solutions are injected via two inlets into the channel, start to mix at junction of two channels, and flow together along the channel. An incomplete mixing of the two species occurs as a result of the obstacles in the intersection region (Figure 4a) which is from resolidified material clogging during ablation on PMMA in air at the junction. In contrast, an efficient mixing of the red and blue pigments occurs as shown in Figure 4b. That is, the metal mask method benefits for greatly reducing resolidification of molten PMMA for promoting mixing. Compared to the photolithography, CO_2_ laser can be a quick and inexpensive method for form the mixing channel. The metal mask also assisted CO_2_ laser ablation, which can effectively solve the traditionally resolidified block problem as above.

**Figure 5:**
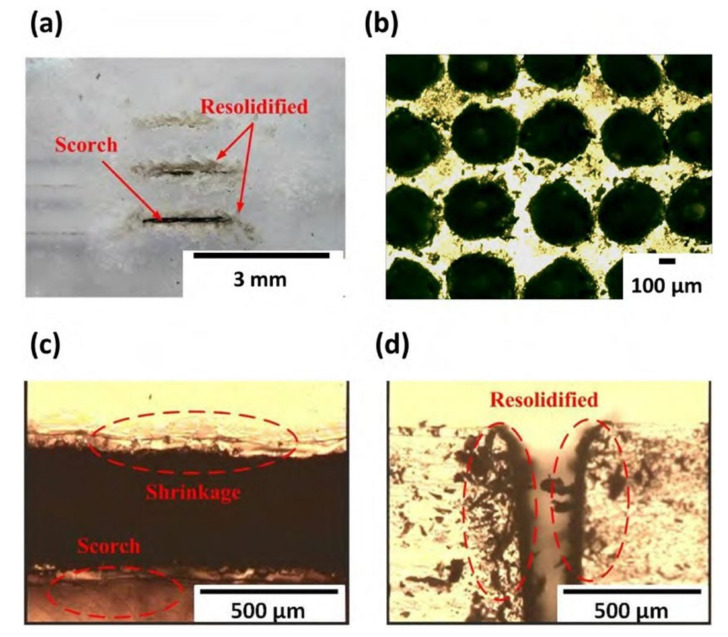
Optical micrographs of the direct CO_2_ laser ablated PDMS channels in air: (**a**) The full view sample of three ablated channels, and (**b**) the ablated holes array, (**c**) the top-view channel rims with the defects of shrinkage and scorch around the rim of channels, and (**d**) the cross-sectional channel with the re-solidified object on the profile.

The optical micrographs (OM) of the directly ablated PDMS channels or holes using direct CO_2_ laser processing in air for one pass are shown in Figure 5. The traditional laser ablated PDMS drawbacks occur clearly. Figure 5a is the full-view image of the channel sample. Figure 5b shows the array of holes with the bulge around the circles’ rims. Figure 5c,d shows the defects of scorches with shrinkage and resolidification formed around the microchannel ablated in air. They are related to the photo-thermal mechanism during ablation without directly breaking chemical bonds. The large temperature gradient easily leads to the distortion of PDMS material during CO_2_ laser ablation through the melting, decomposing, and then vaporizing from the molten material. In conventional Gaussian distribution of the laser energy, it is mostly focused on the center and dispersed around the working area. Figure 5c shows the heat accumulation induced scorches formation and a slight shrinkage at both sides of the channel during laser ablation. Figure 5d displays the debris on the channel edges due to the resolidification of splashing or surface-tension-driven molten polymer by air cooling. So, the thermal defects of scorched, shrunken, and resolidification of the PDMS is easy to form on the surface after laser ablation.

**Figure 6:**
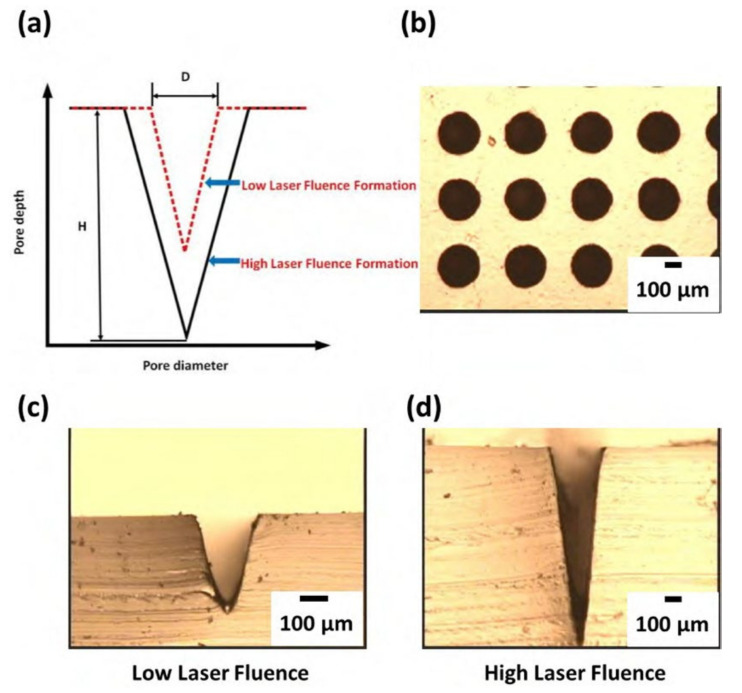
(**a**) Schematic different laser fluence with Gaussian-like distribution for the ablated PMMA profile formation, (**b**) the PMMA holes array formed at 3-W laser power and 114 mm s^−1^ scanning speed for one pass; the cross-sectional optical micrographs of the PMMA mold formed at (**c**) low fluence and (**d**) high fluence.

The problem of direct PDMS ablation in air can be improved using the integration process of the laser ablated PMMA mold together with PDMS casting and/or two-step PDMS casting for dual-tone microstructure conversion. The schematic high and low laser fluences of Gaussian-like distribution are shown in Figure 6a for the ablated profiles of PMMA. The greater ablation depth and wider diameter of the PMMA profile are formed at the higher laser fluence with more input laser energy. The ablated PMMA holes array ablated by direct CO_2_ laser processing in air for one pass at a proper laser parameter is shown in Figure 6b for an average diameter of about 280 μm in a good surface quality. A different featured profile can be fabricated by adjusting the laser power, scanning speed, and pass during ablation. The cross-sectional images of the PMMA microneedles master molds at low and high powers at a constant scanning speed are shown in Figure 6c,d, respectively. The higher laser power results in a higher ablation depth.

**Figure 7:**
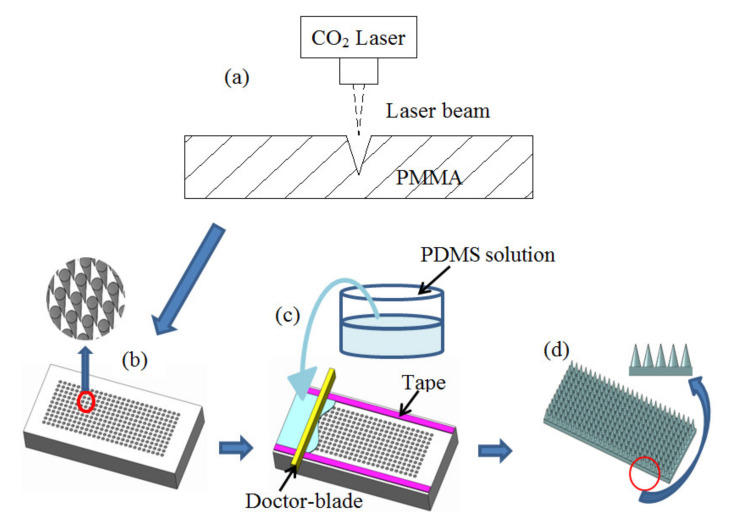
Schematic process flow of the microneedle-PDMS (MN-PDMS) film: (**a**) CO_2_ laser ablated PMMA to produce the MN-hole array, (**b**) the micro-holes array mold and an enlarged image of MN array, (**c**) the PDMS solution poured into the micromold and curing, and (**d**) the PDMS was cooled down to room temperature, peeled off from the mold, and a magnified image of the MN structure from the front.

**Figure 8:**
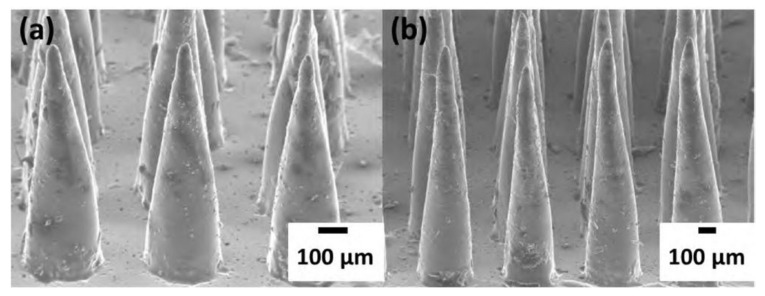
SEM micrographs of the cast PDMS microneedles array from the PMMA mold ablated at laser powers of: (**a**) 3 W and (**b**) 10 W at a constant 114 mm s^−1^ speed.

The schematic process flow of the microneedle-PDMS (MN-PDMS) film is shown in Figure 7. First, the CO_2_ laser is used to ablate PMMA to produce the master mold of MN-hole array (Figure 7a,b). The ablation pattern and laser parameters are designed and controlled by a computer-aided design tool of the CorelDraw software for the desired MNs height, aspect ratio, and density. Then, the PDMS casting is performed by pouring a well-mixed-and-degassed compound solution of wt% 10:1 elastomer (Dow corning Sylgard 184) to curing agent into the PMMA mold and cured in the oven (Figure 7c). At last, the MN-PDMS film is cooled down and peeled off the PMMA mold (Figure 7d). Both examples of the MN-PDMS films formed from the PMMA molds ablated at a constant speed of 114 mm s^−1^ at the powers of 3 W and 10 W are shown in Figure 8, respectively. The one-step casting and demolding method to fabricate the MN-PDMS membrane can be applied for TENG sustainable energy application that is described in detail in chapter 5.

## 3. PDMS Surface Modification for Long-Term Hydrophilicity and 2-Step Dual-Tone Casting for Capillary Microfluidics

In the last few decades, microfluidic chips have attracted much attention to fluid mixing, pumping, and control for the LOC or µTAS application. The developed different types of micropumps for active microfluidics are linked to having complex structures, being time-consuming, and having high-cost fabrication due to the multilayer design. The fluid transport can also be driven by surface tension force that is widely studied because of the merits of power-free operation and simple design. Therefore, the capillary-driven chip has great potential for the fluidic pumping together with passive mixing for extending microfluidic application. A crucial issue for the capillary-driven microfluidic systems is to create the long-term hydrophilic wall of the microchannel in order to effectively actuate various kinds of liquid. The PDMS or SU-8 materials are popular materials and are often used to fabricate the capillary-driven chips. However they are hydrophobic materials and are generally modified by different surface treatment for the hydrophilic surface. Unfortunately, the time-limited hydrophilic property i.e., hydrophobic recovery problem often exists in the modified PDMS or SU-8 surface. For example, the oxygen plasma treated PDMS occurs the hydrophobic recovery in less than one hour. That is, it is the fatal flaw of the PDMS or SU-8 material to be used in the capillary-driven fluidic chip. The glass of intrinsic hydrophilicity is also a popular material for the micorfluidic chip but the complicated processing procedures including cleaning, deposition of etching mask, photolithography patterning, HF etching, holes drilling, and high-temperature fusion bonding are major disadvantages. In this section, the facile approach for long-term hydrophilic surface modification is addressed using the combined O_2_ plasma and PEG coating. The extended two-step casting method is simple and practical for the applications to the capillary pumping and mixing in the microfluidic chips with the assistance of dual-tone PDMS microstructure fabrication through the laser ablated PMMA concave mold.

**Figure 9:**
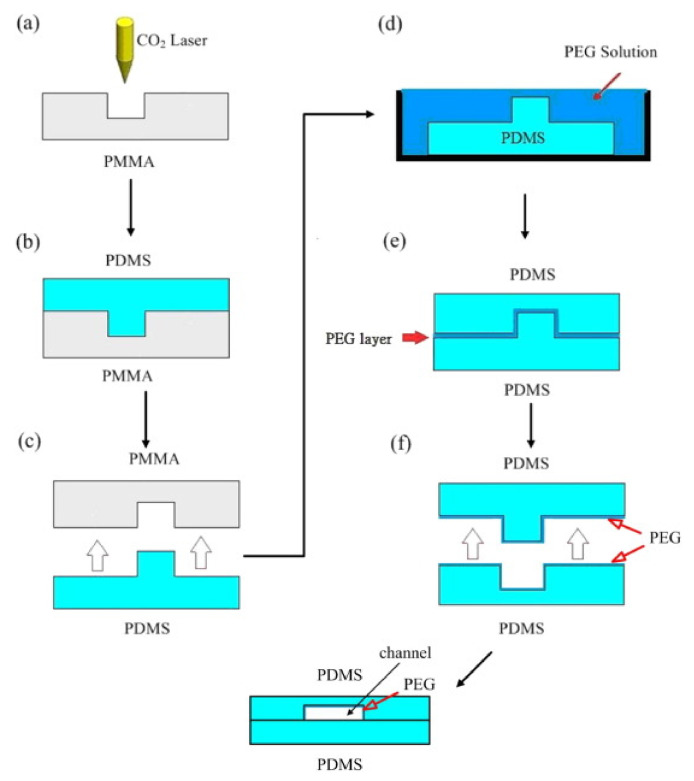
Scheme of fabricating the PDMS microfluidic chip includes: (**a**) the CO_2_ laser ablation, (**b**,**c**) the first-step casting of PDMS, (**d**) surface modification, and (**e**,**f**) the second-step casting.

The scheme of fabricating the all PDMS microfluidic chip using CO_2_ laser ablation, surface modification, and two-step casting is shown in Figure 9. First, the designed microchannel structure is formed on PMMA by a CO_2_ laser ablation (Figure 9a). The well-mixed PDMS solution in a weight ratio of 10:1 (elastomer to hardener) was placed in a vacuum pump to remove any air bubbles, poured onto the PMMA master mold, cured at 70 °C for 30 min, and cooled to ambient temperature (Figure 9b). Then a PDMS convex mold was stripped from the PMMA mother mold (Figure 9c). The O_2_ plasma was used for surface modification of the PDMS mold for 1 min and then the mold was immersed in a PEG solution (Figure 9d). The PDMS convex mold was used for the second casting following the steps in Figure 9b,c to form the PDMS concave microstructure and the bonding with a flat PDMS to form the microfluidic chip (Figure 9e,f and the final chip). The integrated process for all PDMS microfluidic chips is much faster and cheaper compared with the conventional photolithography-based technology. In order to assess the long-term hydrophilic behavior measured by surface contact angle, three kinds of surface treatment method are applied, that is, the O_2_ plasma, the PEG coating, and the O_2_ plasma followed by PEG (O_2_-plasma-PEG) coating before the chip bonding. The PDMS sample activated by O_2_ plasma is performed using a 30 W RF power at 500 mTorr for 60 s. The PDMS sample coated with a PEG film is dipped in a PEG solution of 0.1 M for 25~30 min.

**Figure 10:**
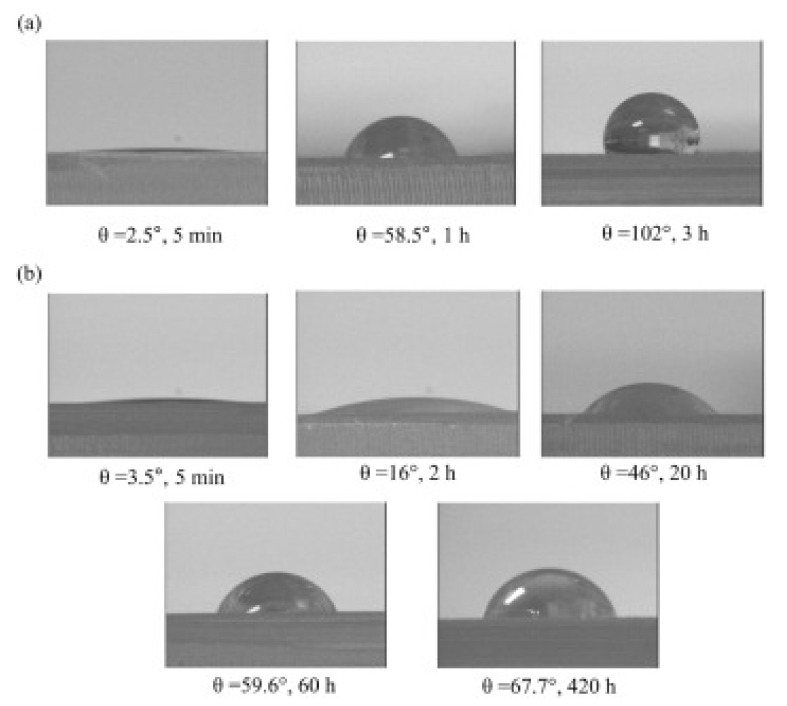
Optical micrographs of the contact angle of a DI water droplet on the PDMS substrate: (**a**) 5 min, 1 h, and 3 h after pure O_2_-plasma treatment; (**b**) 5 min, 2 h, 20 h, 60 h, and 420 h after O_2_-plasma-PEG treatment.

The property of a PDMS surface modified by the O_2_-plasma and O_2_-plasma-PEG treatment for various times is examined by contact angle measurement with DI water droplets for the retained hydrophilic duration as shown in Figure 10a,b, respectively. Pure PDMS is intrinsically hydrophobic with a contact angle of 100° ± 3°. Pure O_2_-plasma treatment on PDMS can initially produce a superhydrophilic surface with the contact angle of 2.5° after 5 min, to be 58.5° after 1 h and becomes hydrophobic to 102° after 3 h (Figure 10a). The hydrophobic recovery of the O_2_-plasma-PDMS sample occurs after a short time of retainment, although the O_2_ plasma may create the oxygen functional groups initially on the PDMS surface for super hydrophilicity with great Si–OH bonds. Regarding the PDMS with pure PEG dip coating, the hydrophilic behavior is enhanced longer to 24–48 h compared to the O_2_-plasma-PDMS sample. The uniformity coating is a challenge for the pure PEG coating. If combining both the O_2_ plasma and PEG coating on the PDMS, the hydrophilicity behavior can be tremendously kept even to 420 h with a contact angle less than 70° (Figure 10b). It implies that the PDMS surface with O_2_-plasma treatment may greatly promote the adhesion of PEG coating on PDMS. The surface treatment method affects the adhesion behavior and surface property. It is a good opportunity for O_2_-plasma PEG-treated PDMS for self-driven capillary microfluidic devices and/or LOC applications.

**Figure 11:**
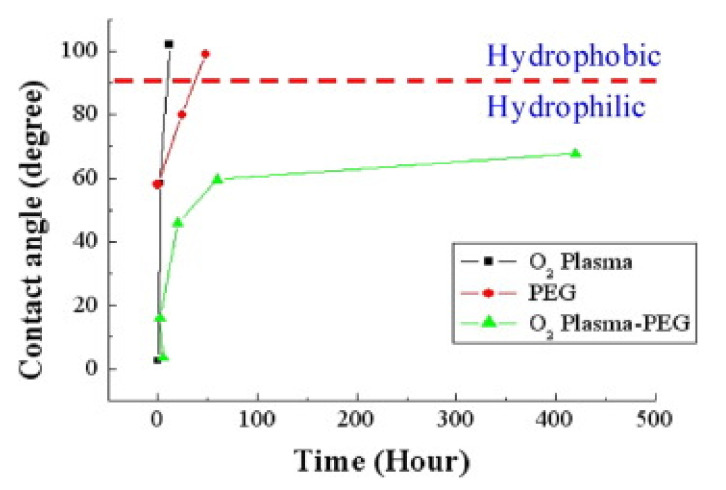
The contact angle is a function of the retaining time of PDMS after different treatment methods i.e., the O_2_ plasma, the PEG, and the O_2_-plasma-PEG modification.

The evolution of quantitative transition from the hydrophilicity to hydrophobilicity of treated PDMS is examined from the relationship between the contact angle and retaining time as shown in Figure 11. Three different methods, that is, the O_2_ plasma, the pure PEG, and the combined O_2_-plasma-PEG modification are compared. The transition point is set at a 90° contact angle. The hydrophobic recovery effect of the O_2_ plasma-PDMS sample exhibits the contact angle larger than 90° by 2 h. Moreover, the contact angle of the PEG-PDMS sample shows that the contact angle increases to 80° after 24 h and becomes larger than 90° at about 38 h. The O_2_-plasma-PEG-PDMS samples retain good hydrophilicity with a contact angle of 67.7° after 420 h, i.e., 17.5 days. Therefore, the O_2_-PEG modification appears to be an excellent method for long-term hydrophilicity applied to the capillary-driven microfluidic chips. The microstructure and bonding behavior of O_2_-plasma-PEG-PDMS may be examined by the FTIR spectra of the sample stored in a different time from 1 h to 420 h after modification with the reference sample of pure PDMS [87]. The absorption peak at 2880 cm^−1^ of FTIR is related to the existence of -CH_2_O- groups of PEG compared to the pure PDMS. The absorption peaks at 1338 cm^−1^ and 1106 cm^−1^ are for the -CH_2_O- groups and C–O–C groups of PEG. The PEG-related absorption peak intensity gradually decreases with increasing retaining time and is still clear after 420 h. This indicates the O_2_-plasma-PEG treatment on the PDMS surface is practical for a long-term hydrophilic effect.

**Figure 12:**
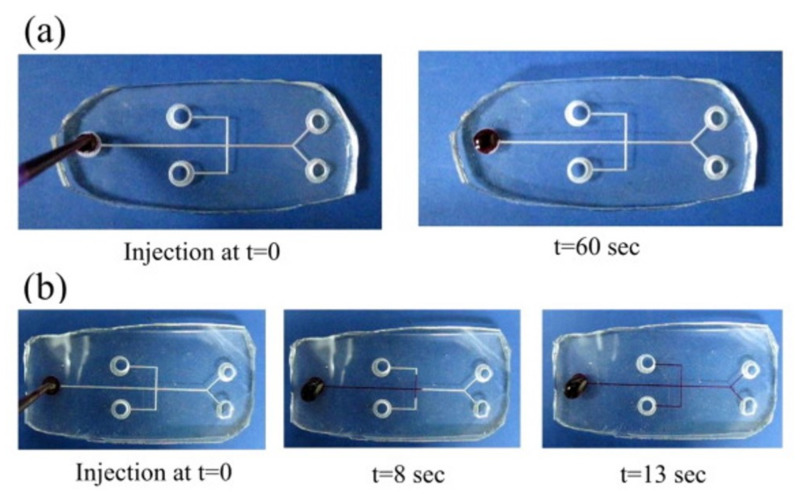
The capillary flow test with injection of Rhodamine B dye into the PDMS microchip: (**a**) 2 h after O_2_ treatment, and (**b**) 420 h after O_2_-plasma-PEG treatment. The former shows no capillary flow of dye into the microchannel after 60 s while the latter exhibits the capillary flow of dye to half of full channel after 8 s, and through the microchannel after 13 s.

The capillary-driven flow test is performed using the Rhodamine B dye injected into the two PDMS microchips 2 h after O_2_ plasma treatment and 420 h after O_2_-plasma-PEG treatment, respectively, as shown in Figure 12a,b. There is no capillary flow of Rhodamine B into the microchannel of the O_2_ plasma-PDMS chip after 60 s (Figure 12a) while the O_2_ plasma-PEG-PDMS chip clearly exhibits the capillary flow to half of the full channel after 8 s, and completely through the microchannel after 13 s (Figure 12b). The mean flow velocity of Rhodamine B calculated is about 3.85 mm/s. This manifests that the hydrophobic recovery occurs in the O_2_-PDMS chip after 2 h but the O_2_-PEG-PDMS chip retains the hydrophilicity property for 420 h and more. It is in good agreement with the contact angle measurement and microstructure analysis. The long storage of the hydrophilic O_2_-PEG-PDMS chip still makes liquid flow smoothly. This indicates that the capillary microfluidic system may be realized using this long-term hydrophilic modification for the O_2_-PEG-PDMS chip. For capillary flow of two fluids to mix in the microchannel, the trigger valve design is important for the fluid flow with delay regarding the wait for the other to merge toward the mixing unit [88].

## 4. Design and Performance of PDMS-Based Microfluidic Chips for High-Efficiency Micromixers and Nanoparticles Synthesis

### 4.1. The Capillary Micromixer with Meander Microfluidic Channel

**Figure 13:**
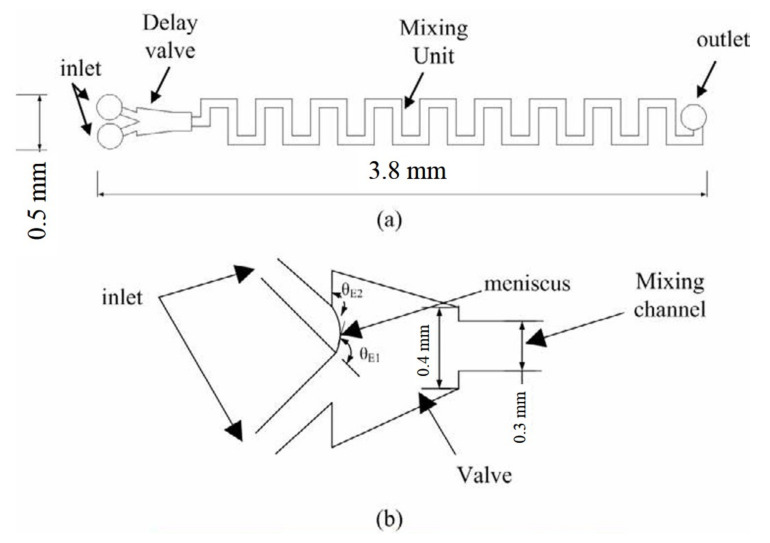
Schematic diagram of the designed: (**a**) Meander chip and (**b**) the triggering valve.

Figure 13 shows the schematic diagram of the designed meander micromixer with an open surface, a triggering valve, and nine mixing units with a chip size of 50 mm (L) × 20 mm (W). The triggering valve is crucial for the capillary micromixer. An important parameter of the expansion angle is defined as the opening angle in the triggering valve. The fluid cannot flow forward if the merged expansion angle and contact angle is larger than 90°, that is, the stop-valve effect. In contrast, the so-called delay-valve effect occurs to decrease the fluid speed within the channel as the expansion angle is larger than 0°, combined with a contact angle less than 90°. It is noted that the delay valve effect makes the injected fluid unable to advance forward without meeting the other fluid at the triggering valve. The design of triggering delay-valve is important for the arrival of two fluids before mixing without the backflow problem. The surface tension of fluid on the channel wall of the valve decides the contact angle in the valve that dominates the capillary flow in the microchannel. In addition, whether the fluid advances in the channel or not is affected by the capillary pressure in a channel.

**Figure 14:**
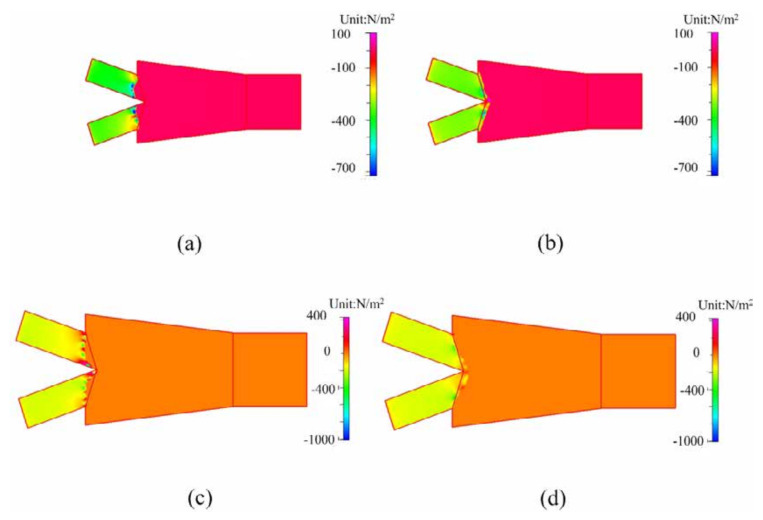
The CFD-ACE simulation results of valve pressure difference of the closed channel (**a**,**b**) and the open-surface channel (**c**,**d**) before and after the triggering effect.

The difference of capillary pressure in the triggering valve before and after the triggering effect is simulated by CFD-ACE software. The simulate results in both the closed and open-surface channels are shown in Figure 14. For the closed channel of 250 μm before the triggering, the capillary pressure is approximately −350 Pa (N m^−2^) within the inlet channel to impede one fluid from advancing forward as shown in Figure 14a. As two fluids merge, the pressure increases to 70 Pa and result in the flow of fluids into the mixing channel as shown in Figure 14b. Furthermore, for the open-surface channel of 130 μm, the pressure before merging is approximately −200 Pa (N m^−2^) as shown in Figure 14c and enhanced to approximately 40 Pa after triggering as shown in Figure 14d. That is, the triggering effect on the capillary pressure in the open-surface microchannel is lower than the closed channel. This indicates that the open-surface microchannel can use a lower capillary pressure to actuate fluid flow than the closed channel due to a lack of the upper cover. Another advantage of the designed open-surface channel with the lower capillary pressure difference is to decrease the bubble formation during the fluid flow due to the flow velocity discontinuity from the varied angle, width, and resistance with surface morphology. Lower flow resistance from smaller pressure difference may reduce the formation of bubble nuclei at the channel intersect and corner of the valve. In other words, the open-surface channel of three walls results in a lower difference of capillary pressure for a lower probability of bubble formation compared to the closed channel of four walls leads. Using the triggering-delay valve and open-surface microchannel design can promote the problems of backflow and bubble formation due to the reduced pressure difference within the channel.

**Figure 15:**
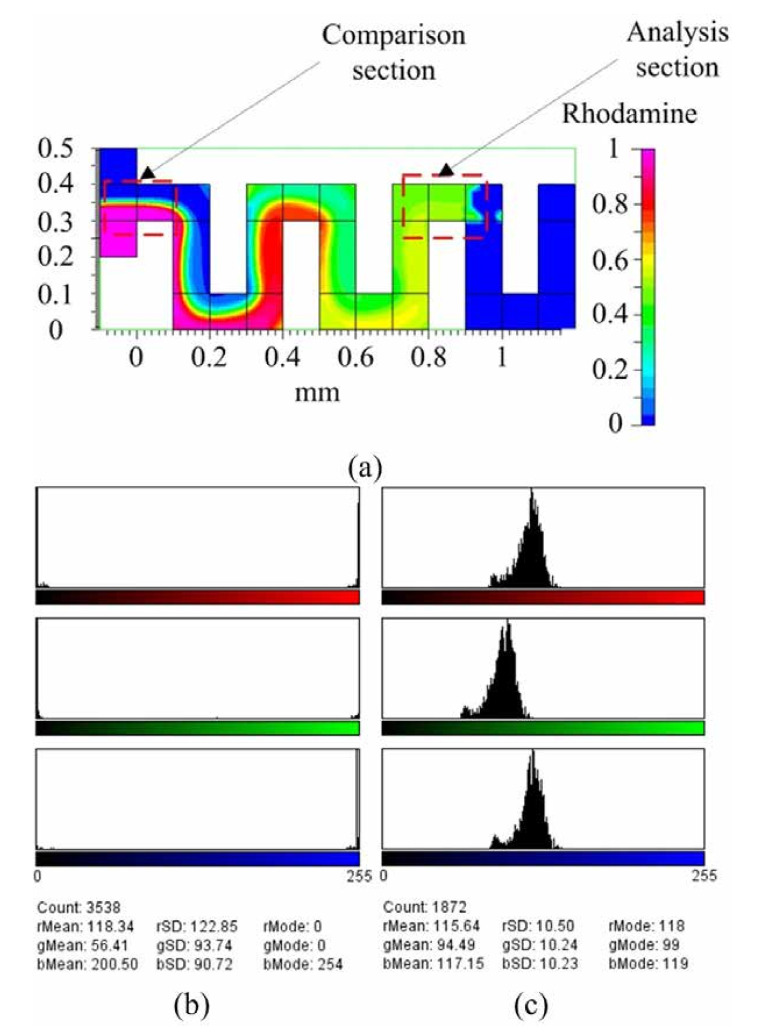
The CFD-ACE simulated mixing extent of the meander micromixer at: (**a**) Comparison position and analysis position, (**b**) RGB concentration at comparison position, and (**c**) RGB concentration at analysis position.

The capillary flow occurs at a low Reynolds number that is the laminar flow and the diffusion dominates mixing efficiency. The diffusion mixing in a straight channel needs a long time for efficient mixing. However, the meander microchannel with the geometry advantage of increasing the contact area with a folding and stretching effect through each corner can greatly enhance the fluidic mixing in a short time. The CFD-ACE simulated mixing extent of the meander micromixer is shown in Figure 15. The variation of intensity of the blue and red dyes in the mixing unit is analyzed by ImageJ software. Figure 15a shows the capture points at the mixing front for the comparison section and at the end for the analysis section of the valve. Figure 15b,c shows the detailed RGB concentration at the comparison section and the analysis section, respectively. The calculated mixing efficiency increases from about 43% (the mixing front) to 92% (the mixing end).

**Figure 16:**
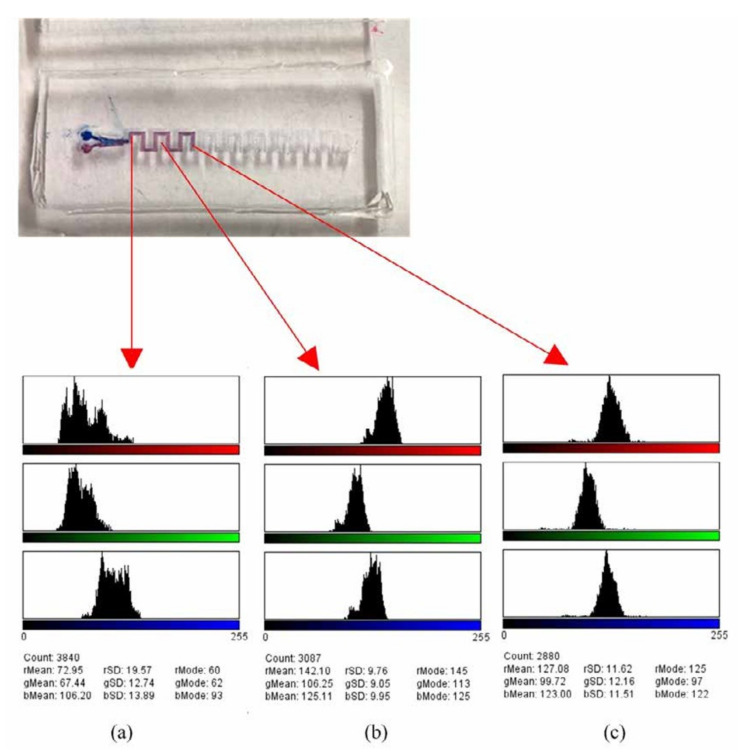
The experiment and analysis of the capillary-driven meander mixer at the: (**a**) Front, (**b**) mid, and (**c**) end of the fluidic flow.

Figure 16 shows the results of experiment and analysis of the capillary-driven meander micromixer using the computer graphic analysis software for the color change of concentration from the mixing front, to the mid, to the end. For example, the red color concertation from the front to the end of mixing channel changes from 72.95 (the front) to 142.10 (the mid) to 127.08 (the end) of 255. The meander microchannel enhances the mixing through two mechanisms of diffusion and the fluid rotation due to the baffle effect of the channel. For the evolution of blue color concentration from the front to the end of the mixing unit, it is from 106.20 (the front) to 125.11 (the mid) to 123.00 (the end) of 255. It indicates that both the diffusion and rotation effect of fluid enhances the mixing efficiency from 57% to 96%. It is in good agreement with the simulation results.

### 4.2. The In-Plane Rhombic Micromixer

**Figure 17:**
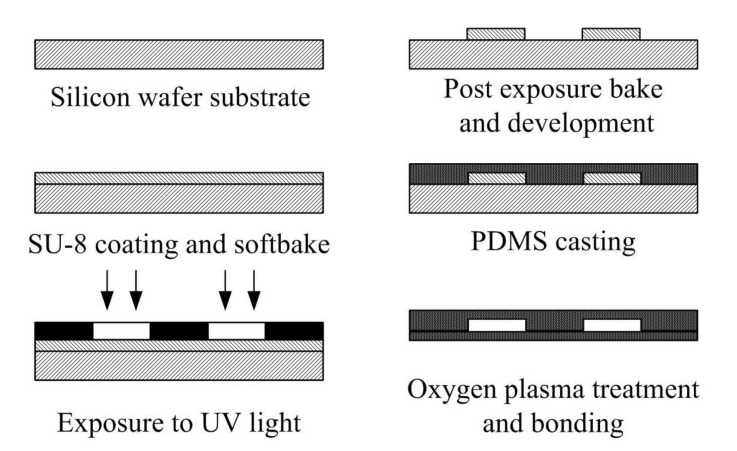
Fabrication processes of the planar PDMS micromixers. Major processes are photography, casting, and oxygen plasma bonding.

All fabrication processes of the PDMS-based micromixers are described and shown in Figure 17. Three major processes, photolithography, casting, and oxygen plasma bonding, were used to fabricate the PDMS-based micromixers.

A silicon-based master mold is needed for replication of the microchannel geometry. First, SU-8 photoresist (SU8-50, MicroChem Corporation, Westborough, MA, USA) was coated on the 4” silicon wafer by a spin coater (MSC 300, Tekstarter Co., Hsinchu, Taiwan). The desired photoresist thick of the micromixer was 130 µm. Then, SU-8 coated silicon wafer was soft baked at 95 °C for 40 min. Then, SU-8 exposure by a standard UV-light mask aligner (500-IR, Optical Associates Inc., Milpitas, CA, USA). For the specific thick of the photoresist film, an exposure dose of 300 mJ/cm^2^ is required at a 365-nm wavelength. Following exposure, post-exposure bake was performed to cross-link the photoresist layer at 95 °C for 50 min. After the process of the post-exposure bake, the coated silicon wafer was immerged into SU-8 development solution for approximately 30 s. Then, this silicon wafer was cleaned with deionized water and dried with nitrogen gas.

**Figure 18:**
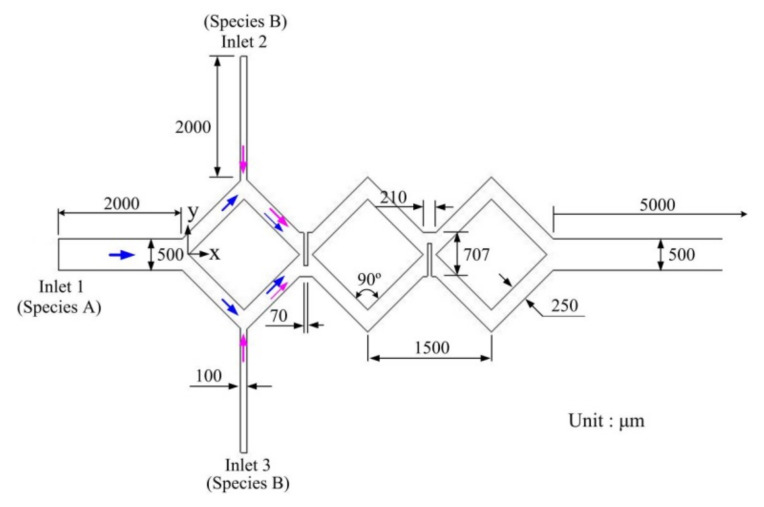
Schematic diagram of the modified rhombic micromixer with a turning angle of 90° and two baffles. Different gap ratios were considered to investigate the mixing efficiency.

The in-plane rhombic mixers, baffles between two rhombi are designed to create a planar recirculation and stretching effect for enhancing fluid mixing. In order to investigate the baffle effect, a combination of three rhombi, turning angle of 90°, and 250 μm in a rhombic-channel width was adopted in the absence of the nozzle. Figure 18 shows the schematic diagram of the rhombic micromixer with baffles. Gap ratio is defined as the gap size divided by the whole width (707 μm).

In order to investigate the degree of fluid mixing, mixing efficiency can be calculated by the expression:

(1) $$M=1-\frac{\sqrt{\frac{1}{N}\sum_{i=1}^{N}{\left(k_{i}-\bar{k}\right)}^{2}}}{\bar{k}(1-\bar{k})}$$

where *M* is the mixing efficiency, *N* is the total number of points, and *k_i_* is the mole fraction distribution over the whole cross section and the average molar fraction. Value of mixing efficiency ranges from 0 (0% mixing) to 1 (100% mixing).

**Figure 19:**
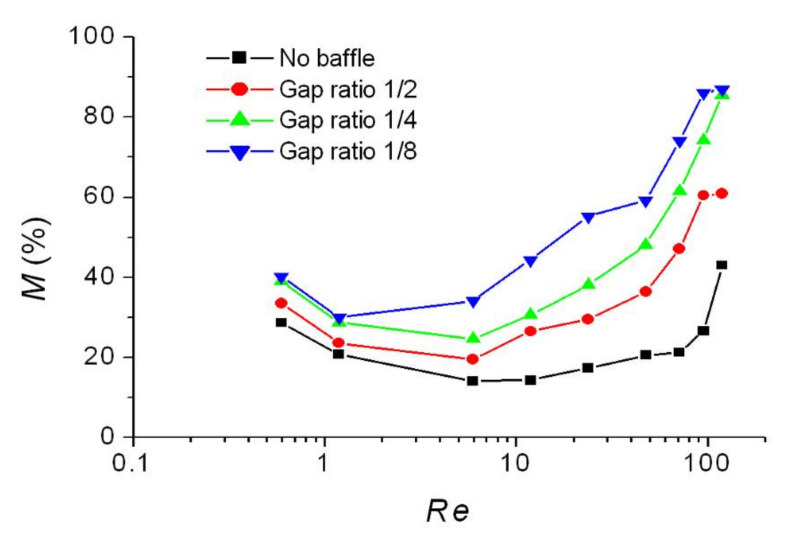
Mixing efficiency of the modified rhombic micromixer with different gap ratios as a function of Reynolds number.

The three-rhombus micromixers with different gap ratios (1/2, 1/4, and 1/8) were investigated at different Reynolds numbers (*Re*). Figure 19 shows the mixing efficiency of the three-rhombus micromixers with different gap ratios as a function of Reynolds number. Variation in mixing efficiency also shows two mixing regions, diffusion region, and convection region. In the diffusion region, an increase in mixing efficiency with a decreasing gap ratio is not very obvious. For example, the mixing efficiency of the rhombic micromixer without baffles is 28.7% at *Re* 0.595. For gap ratios of 1/4 and 1/8, the mixing efficiency increases from 28.7% to 39.1% and 40.2% because of baffle constriction.

**Figure 20:**
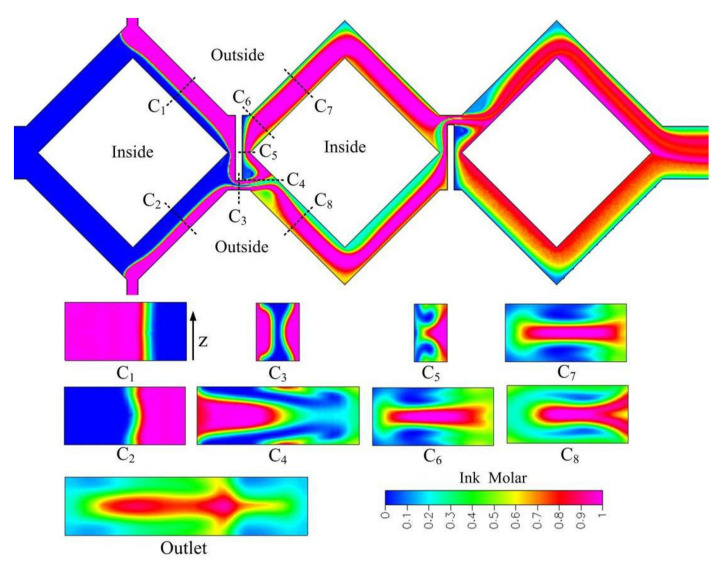
Cross-sectional concentration distributions of the three-rhombus micromixer with a gap ratio of 1/8 at *Re* 23.8 (x-y cross section is at half-depth plane).

As gap ratio is decreased from 1/4 to 1/8, focusing and stretching effects will be stronger. Figure 20 shows concentration distributions of the cross sections C_1_-C_8_ and outlet of the rhombic micromixer with a gap ratio of 1/8 at *Re* 23.8. Resident time and distorted interfaces in cross section C_3_-C_8_ are similar between a gap ratio of 1/4 and 1/8. Due to stronger focusing effect, better mixing in cross section C_5_, C_7_, and C_8_ can be obtained compared with a gap ratio of 1/4. In addition, improved mixing can be obtained at the outlet.

**Figure 21:**
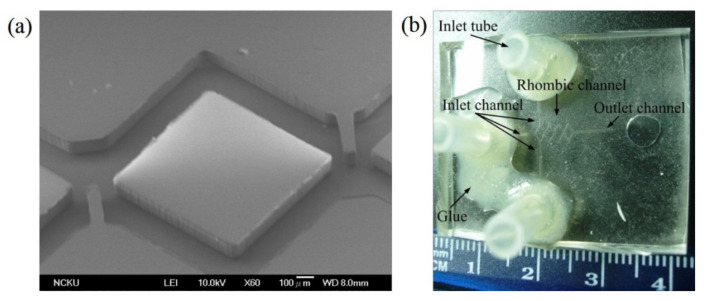
(**a**) SEM image of the PDMS replica layer of the rhombic micromixer with a gap ratio of 1/2. (**b**) A finished PDMS micromixer with three inlets and one outlet.

**Figure 22:**
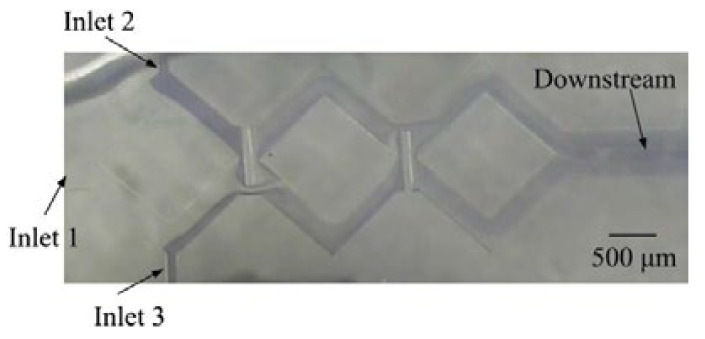
Optical micrographs of fluid mixing in the rhombic micromixer with a gap ratio of 1/8 at Re = 20. The top view image is recorded by a Charge-coupled Device (CCD) camera.

SEM image of the replicated PDMS channel layer before bonding is shown in Figure 21a This SEM image shows two baffles were added into the junction between two rhombi. Silicon tubes were used and glued to the PDMS-based micromixer, as shown in Figure 21b. There are three inlets and one outlet in this rhombic micromixer. The optical image of experimental fluid mixing in the rhombic micromixer with a gap ratio of 1/8 at *Re* 20 is shown in Figure 22. Increasing the Reynolds number to 20 results in much better mixing at the second and third rhombic channels.

### 4.3. The Obstacled Micromixer and Its Application for Nanoparticles Synthesis

**Figure 23:**
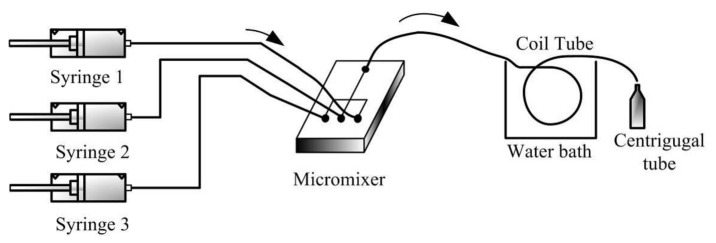
Continuous-flow system with a planar micromixer and coil aging tube.

A continuous-flow synthesis system is shown in Figure 23. An obstacled micromixer was further applied to synthesize silica nanoparticles at different recipes and temperatures. The channel structure with a gap ratio of 1/8 and three mixing units was adopted due to the good mixing efficiency. Two stock solutions were loaded into different syringes, and equal flow rates were pumped into the micromixer by using two syringe pumps (KDS 200, KD Scientific Inc., Holliston, MA, USA). The solution of tetraethoxysilane (TEOS) in ethyl alcohol was supplied to the main inlet of the obstacled micromixer. A solution of ammonia, water, and ethyl alcohol was applied to side inlets of the obstacled micromixer. Inlet ports of the obstacled micromixer were interfaced with three syringes using silicon tubes. An aging channel with 4 m in length was connected with the outlet port of the obstacled micromixer.

**Figure 24:**
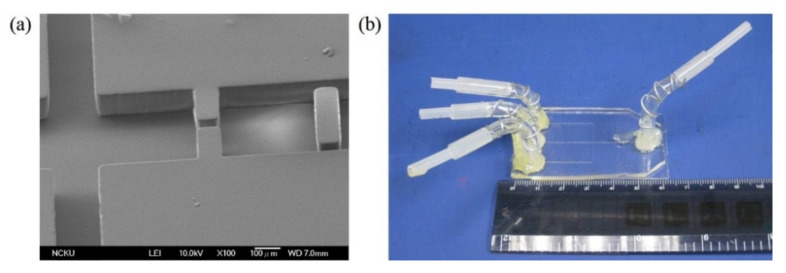
(**a**) SEM image of the PDMS replica layer with gap ratio of 1/8. (**b**) An obstacled micromixer with three inlets and one outlet.

Beside the fabrication of the rhombic micromixers, an obstacled micromixer had also been fabricated by using the same fabrication process and parameters. One-step lithography, PDMS casting, and oxygen plasma-bonding techniques were used to fabricate the PDMS-based obstacled micromixers. Figure 24a shows the SEM image of the replicated PDMS layer with a gap ratio of 1/8. This replicated PDMS channel layer was further bonded with another PDMS layer by using oxygen plasma treatment. In order to increase the bonding strength and avoid liquid leakage, a modification time of 30 s was performed. After that, silicon tubes were glued with three punched inlet holes and one outlet hole in the PDMS-based micromixer, as shown in Figure 24b.

**Figure 25:**
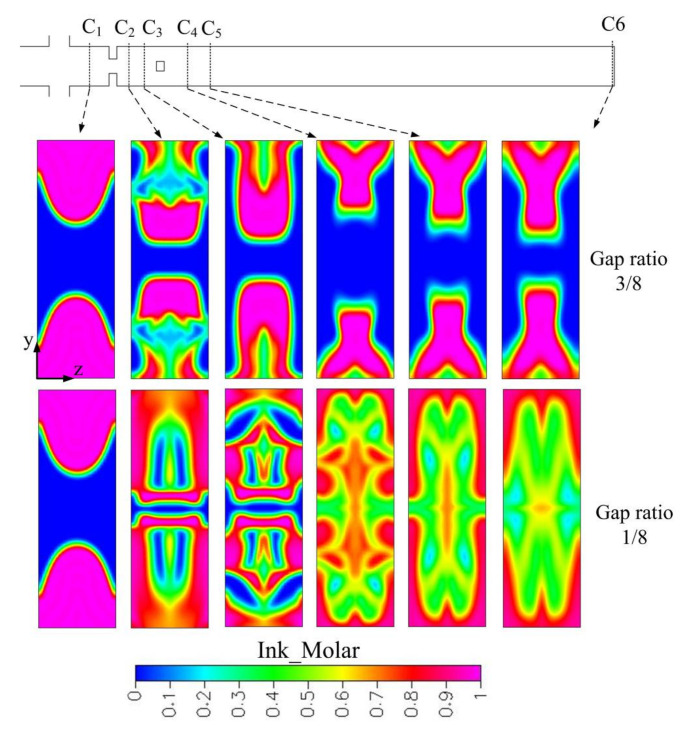
Cross-sectional concentration distributions of obstacled micromixer with one mixing unit at different gap ratios and *Re* 60 (obstacle width 80 μm).

**Figure 26:**
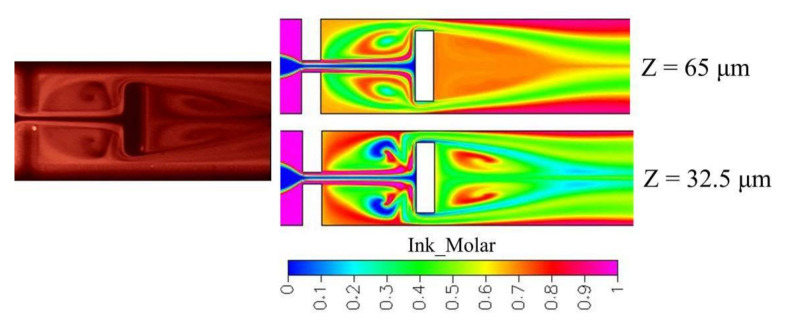
Mixing comparison between experiments and simulation at *Re* 60. Experimental top-viewing images were captured by the conventional fluorescence microscope.

Obstacles with different sizes were added into the cross-shaped microchannel for exploring flow behaviors and mixing efficiency. Stronger and larger recirculations are created to agitate two species within the mixing chamber. However, at the same Reynolds number of 60, only smaller recirculations are created in the micromixer with a gap ratio of 3/8. A detailed mixing process of the micromixers with different gap ratios is shown in Figure 25. For a gap ratio of 1/8, complex interfaces are created because of convection effect. However, interfaces stretch slightly in the micromixer with a gap ratio of 3/8 and little mixing is enhanced. A stronger recirculation effect in the micromixer with a gap ratio of 1/8 can also be observed by experiments, as shown in Figure 26. Experimental top-viewing images were captured by the conventional fluorescence microscope. At a high Reynolds number, such as *Re* 60, an obvious recirculation effect is created because of high flow velocity.

**Figure 27:**
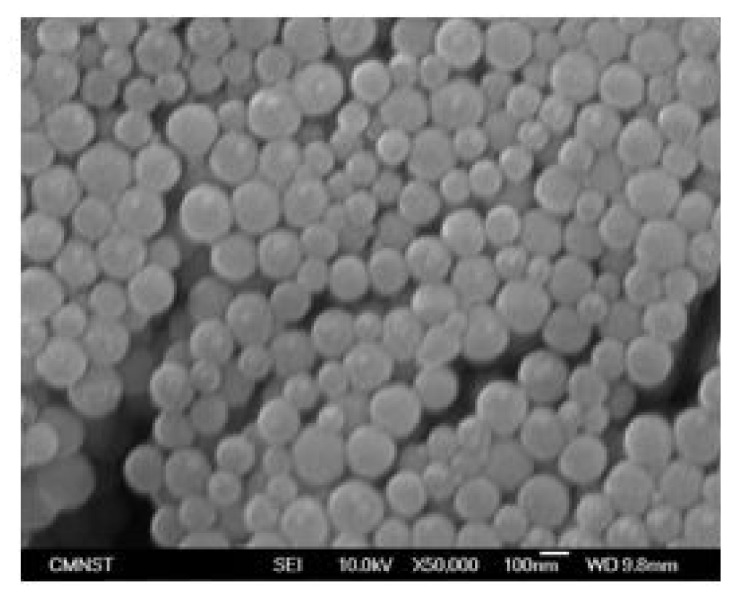
The SEM image of silica nanoparticles with an average diameter about 140 ± 20 nm at a high flow rate.

This high-efficiency obstacled micromixer was further applied to nanoparticle synthesis. Its corresponding Reynolds number is about 60, and two streams can be mixed by molecular diffusion by means of the solution listed in the recipe of Table 3. Figure 27 shows the SEM micrograph of the synthesized silica nanoparticles with an average diameter about 140 ± 20 nm. The synthesis result shows silica nanoparticles can be synthesized using this obstacled micromixer with a short mixing length at *Re* 60.

## 5. Design and Performance of Al/PDMS TENG for Sustainable Energy Application

**Figure 28:**
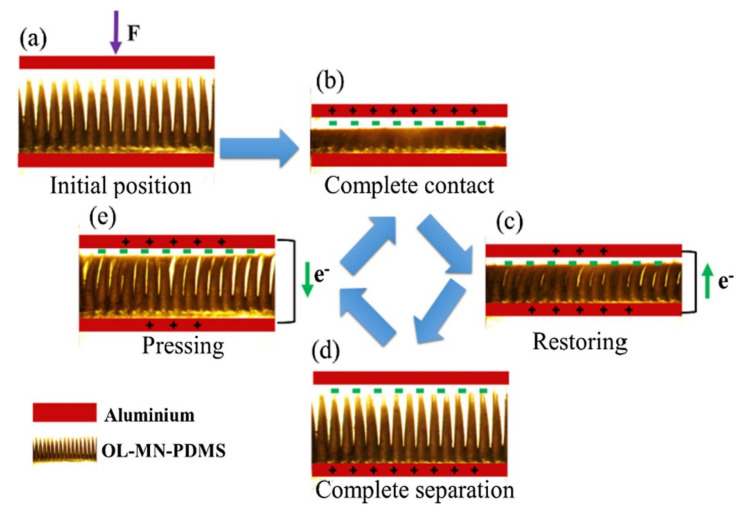
The basic operating mechanism of the overlapped microneedle (OL-MN)-TENG: (**a**) Initial position, (**b**,**c**) complete contact and restoring with reducing force and (**d**,**e**) complete separation and pressing with increasing force. Then cycling operation through (**b**–**e**).

Figure 28 shows the basic operating mechanism of the triboelectric nanogenerator (TENG) for charging occurrence on triboelectric materials. The alternating current is generated by TENG under the cycling contact-separate operation of two triboelectric material surfaces like PDMS and Al. When the triboelectric materials are pressed to contact and rub with each other, the PDMS induces negative triboelectric charges and inductive positive charges from the Al foil concentrated on contact surfaces, separately. After that, the potential imbalance between two tribo-materials is caused by the separation. Therefore, to return electrical equilibrium (Figure 28a) a flow of electrons via an external circuit moves forth and back between the two electrodes. When the hand-pressing force is on the top electrode, the electrode contacts and rubs against the surface of friction materials such as PDMS, which leads to the negative charge on the PDMS surface, and causes the positive charge on Al because of electrical neutrality. The triboelectricity distribution is generated between the two tribo-surfaces of material (Figure 28b). The two electrodes separate with each other under elastic force of springs to release the external force. Thus, the imbalance potential causes a negative current transfer from the top electrode to the bottom through an external circuit (Figure 28c). Therefore, the electrons movement establishes a new electrical equilibrium state (Figure 28d). The reversed movement of electrons from the bottom electrode to the top electrode for a new triboelectric equilibrium state produces the external positive current as the force is applied again (Figure 28e). This new triboelectric equilibrium will reach the full contact state of the two tribo-material surfaces (Figure 28b). Therefore, the cycling operation of the TENG continuously generates the alternating current through an external circuit.

Two key issues of the materials’ triboelectric polarity and the surface morphology for contact area are significantly related to the output performance of TENG. Table 2 lists the comparison of various surface microstructures studied by different researches. The morphology affects the V_oc_ and I_sc_. Increasing the effective contact area can produce more charge transfer during friction for enhancing the performance. For example, the trend of output performance of TENG is flat film < line < cube < pyramid. Increasing the effective surface contact area of morphology is a crucial method for improving the output performance and the sensitivity of the force/pressure sensor. The modified micro/nano morphologies structures such as nano pillars and domes, nano patterns, wrinkles, micro pillar, and microneedles are applied to TENG for promoting the performance. To explain in detail in terms of microfabrication, performance, and application, an example of the complex morphology of overlapped microneedles TENG are described.

**Figure 29:**
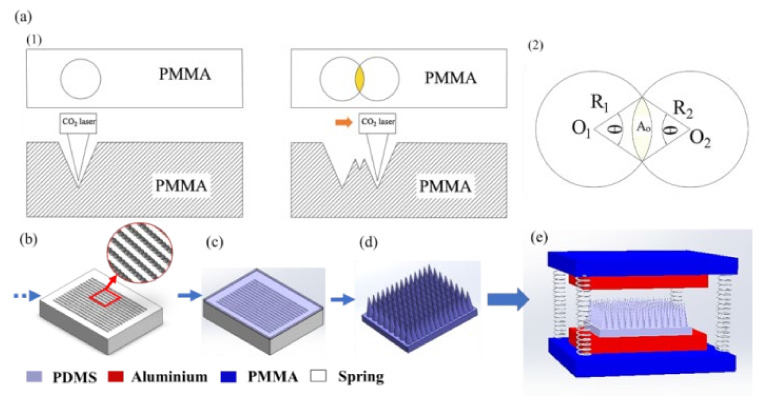
Schematic overlapped two-height microneedle PDMS fabrication and assembly: (**a1**) CO_2_ laser ablated PMMA master mold of two-height holes consisting of a primary height in the laser spot and an extra second-height in the overlapped area; (**a2**) estimation of the overlapped area ratio; (**b**–**e**) Experiment process of PDMS film and the OL-DTH-MN-TENG assembly diagram.

The schematic CO_2_ laser ablated PMMA master mold of two-height holes that is used for casting PDMS two-height microneedles array is shown in Figure 29a1. Figure 29a2 shows the schematic overlapped area Ao (μm^2^) and to estimate the overlapped ratio area, by Equation (2):

(2) $$A_{o}=\frac{1}{2}\left(\frac{\pi \theta }{180}R_{1}^{2}-R_{1}^{2}\sin\theta +\frac{\pi \theta }{180}R_{2}^{2}-R_{2}^{2}\sin\theta \right)$$

where *R_1_* and *R_2_* are the radius of ablated holes in a unit of μm, respectively, and *θ* (º) corresponding to an overlapped angle in a unit of degree. The overlapped microneedle (OL-MN) morphology significantly advances the separated pattern density to increasing the effective surface contact area for high performance. While the single-height OL-MN is used as references, two types of complex two-height microneedle named the overlapped two-height microneedle (OL-TH-MN) and overlapped deep two-height microneedle (OL-DTH-MN) are proposed. The calculated overlap ratio area of OL-MN (245 μm) is 1.5% and the OL-TH-MN (169 μm) is 13.9% following Equation (2). The OL-DTH-MN with an overlapped thermal effect has a larger ablated diameter of holes and the overlap ratio area is 15.4%. Figure 29b–e shows the schematic process of the OL-DTH-MN-TENG.

**Figure 30:**
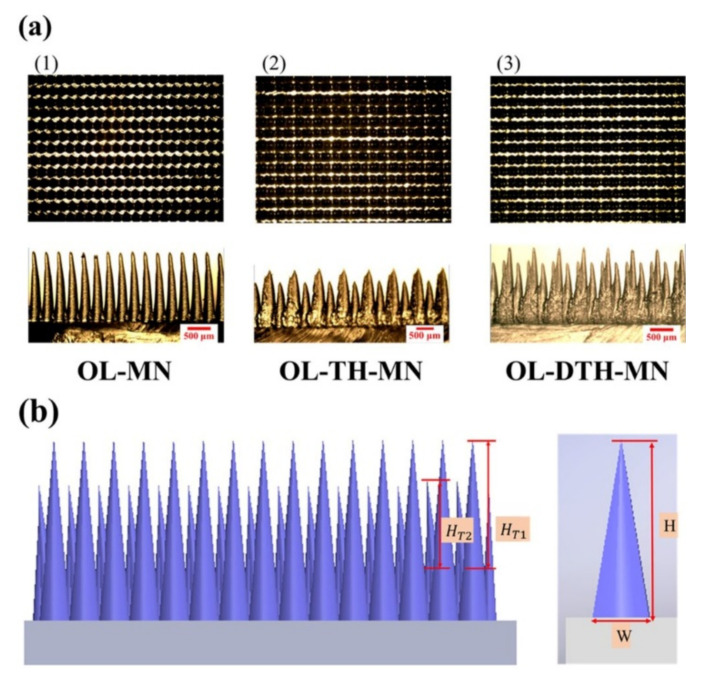
(**a**) OM of the top-view PMMA master mold and the cross-sectional overlapped two-height microneedle PDMS. (**b**) Schematic dimension of OL-TH-MN and OL-DTH-MN.

The OM micrographs of the PMMA master mold (top three) and corresponding cast PDMS microneedles (bottom three) of OL-MN, OL-TH-MN, and OL-DTH-MN, respectively, are shown in Figure 30a. The pattern density of OL-MN is 654 MN cm^−2^ while the higher density of OL-TH-MN and OL-DTH-MN are 965 MN cm^−2^, which is 1.4 times higher than OL-MN. The surface contact area of OL-MN, OL-TH-MN, and OL-DTH-MN is estimated by 3D parametric Solidworks software. The proper overlapped two-height microneedle array highly enhances the contact surface area of the complex deep two-height microneedles array. Moreover, Figure 30b shows that the overlapped area height of the OL-TH-MN and OL-DTH-MN has two kinds of height for microneedles, that is, the overlapped conical tips area above the overlapped bottom area denoted as primary-height HT_1_ and secondary-height HT_2_, and the other is the total height (H) and width (W) of single microneedle including the non-overlapped and overlapped area. The magnitude of H and W of MN is OL-MN > OL-TH-MN but the pattern density of OL-TH-MN (965 MN/cm^2^) is 1.47 times OL-MN (654 MN/cm^2^) due to the contributed laser energy per spot that decreases at shorter ablation spacing. The H and W of OL-DTH-MN is the highest of a single MN due to the increased laser power. The calculated total effective surface area order is OL-DTH-MN (29.69 × 103 mm^2^) > OL-TH-MN (24.38 × 103 mm^2^) > OL-MN (22.91 × 103 mm^2^). It is noted that the OL-DTH-MN enhances the MN height and width to promote the total contact surface area.

**Figure 31:**
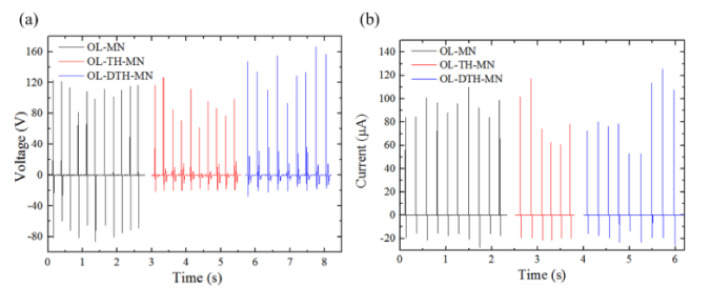
The measured output performance of open-circuit voltage and short-circuit current of overlapped two-height microneedle array: (**a**) Voltage and (**b**) current was measured of the OL-MN, OL-TH-MN, and OL-DTH-MN during real-time hand tapping.

The morphology of the friction layer strongly influences the output performance of TENG. The measured electrical signals of the Voc and Isc of OL-MN, OL-TH-MN, and OL-DTH-MN, respectively are shown in Figure 31. The peak Voc and Isc of OL-MN is 123 V and 109.7 μA, corresponding to the current density Jsc of 3.6 μA/cm^2^. The Voc and Isc of OL-DTH-MN is 167 V and 129.3 μA, corresponding to Jsc of 4.3 μA/cm^2^, while the Voc and Isc of OL-TH-MN is 127 V and 117.6 μA, corresponding to Jsc of 3.9 μA/cm^2^. In comparison, the Voc and Isc of TENG follows the ascending effective surface area order of OL-MN < OL-TH-MN < OL-DTH-MN. The total effective contact surface area is related to the output performance. The more MN number will increase the deformed effective contact area for enhancing the triboelectric charge density. The total effective surface area of OL-DTH-MN is highest at 29.69 × 103 mm^2^ to generate greater charge density on its surfaces area than the flat and OL-MN PDMS films. It gains considerable potential for high-performance TENG application.

**Figure 32:**
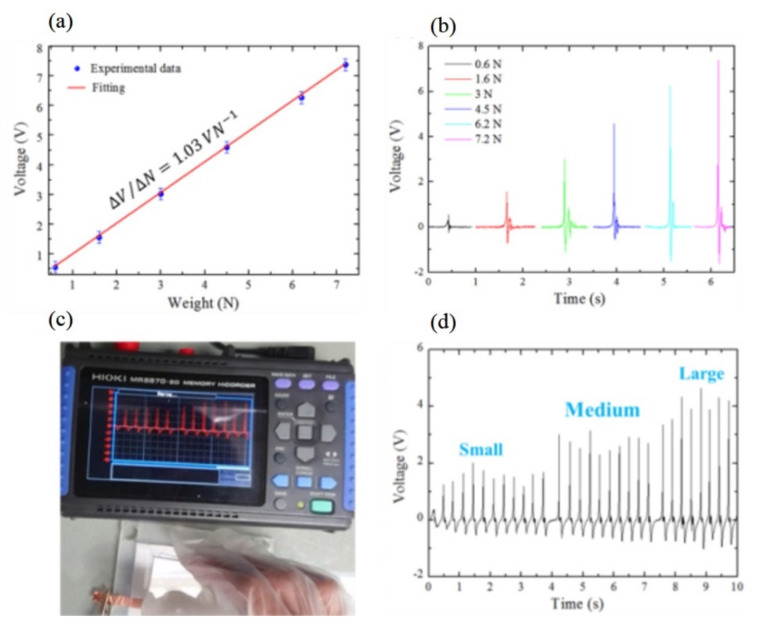
The OL-DTH-MN applied to the weight and pressure sensors: The output voltage of sensors as a function of (**a**) weight; (**b**) the output voltage under a wide range of load weights of 0.6 to 7.2 N; verification of the force sensor under three relative zones of load forces namely small (1.96 N), medium (3.05 N), and large (4.5 N) for the corresponding voltage response: (**c**) The image of OL-DTH-MN through external force by continuous hand tapping, and (**d**) the output voltage for estimating the corresponding force.

The output voltage of the OL-DTH-MN-TENG sensor as a function of the weight is shown in Figure 32a. The OL-DTH-MN-TENG has a high linear sensitivity of the force and pressure sensor at about 1.03 V N^−1^. The device is under a wide range of load force from 0.6 to 7.2 N corresponding to 0.2~2.4 kPa. The response output voltage signal of the force sensor under a direct one-touch load force of 0.6 to 7.2 N is shown in Figure 32b. The OL-DTH-MN-TENG sensor with excellent sensitivity is much higher than the conventional TENGs of about 0.18~0.414 V N^−1^, because of the excellent responding output voltage of OL-DTH-MN-TENG with a high pattern density and two-height MNs for a high deformed contact surface area during one-touch contact and separation. High-cost material, multilayer assembly, and fabricated by expensive and complicated lithography-etching, oxygen plasma, and/or chemical solution processes are generally used to produce conventional TENG force/pressure sensors. In contrast, the OL-DTH-MN-TENG sensor uses a simple low-cost CO_2_ laser process and casting with easy access low-cost PDMS and Al triboelectric materials. Figure 32c and d show the recording image and the response output voltages of the OL-DTH-MN-TENG, respectively, by continuous hand tapping. The output voltage corresponding to the calibration curve under small, medium, and large of applied forces can measure the tapping force magnitude. The estimated forces from the maximum voltage peak under different forces are a small force of 1.96 N, medium 3.05 N, and large 4.5 N, respectively. In brief, the OL-DTH-MN-TENG force/pressure sensor has a high sensitivity of about 1.03 V N^−1^. It can be applications for a touch-force identification control such as user security check and human-machine interfaces processing in the future.

**Figure 33:**
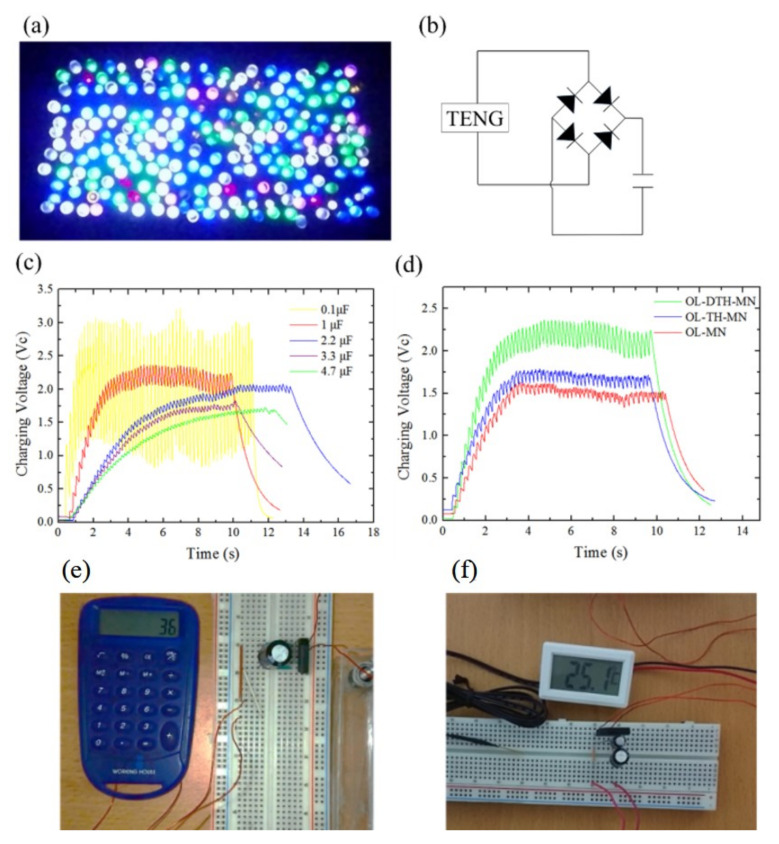
Lighting and energy storage: (**a**) OL-DTH-MN--TENG driving 226 LEDs of six different colors contacted in series, (**b**) circuit design for charging, (**c**) charging voltage of OL-DTH-MN-TENG from 0.1 μF to 4.7 μF capacitor, (**d**) the charging voltage curve on a 1 μF capacitor of OL-MN-TENG, OL-TH-MN-TENG, and OL-DTH-MN-TENG; (**e**) the direct drive of the calculator, (**f**) the self-powered temperature sensor.

Figure 33a shows 226 LEDs of six different colors connected in series lit up by the high-performance OL-DTH-MN. The AC-DC charging circuit design, and the charging voltage of OL-DTH-MN-TENG are shown in Figure 33b and c as a function of time for five different capacitors (0.1~4.7 μF). Furthermore, the OL-MN, OL-TH-MN, and OL-DTH-MN charging behavior are tested for store energy ability on the same 1 μF capacitor for comparison as shown in Figure 33d. The OL-DTH-MN-TENG not only successfully demonstrated the direct drive of the calculator (Figure 33e) but also activate the self-powered temperature sensor to display the ambient temperature (25.1 °C) (Figure 33f).

## 6. Conclusions

PDMS is well-known for its application to various fields, such as microfluidics, biomedical, microneedles, and sustainable energy. Different requirements are concerned with the fabricated microstructure of PDMS and its non-toxic, transparent, flexible, biocompatible, stability with fluidics, negative triboelectricity, and/or hydrophobic properties. The PDMS microfabrication and design for the microfluidics, microneedles, and recent sustainable energy application are reviewed in short and highlighted regarding the aspect of the facile laser micromachining on polymer materials of PMMA and PDMS to produce the desired microstructure and the mother mold for the following one-step and two-step casting method to duplicate the dual-tone convex and concave structures for different applications according to the featured specification of devices. Compared with traditional Si and glass microfluidic chips using the photolithography, etching, and deposition processes, the laser micromachining has advantages of being simple, fast, and direct-write for different geometrical shapes. The CO_2_ laser ablation-induced defects such as bulges, splashing, resolidification, and scorches around the microstructure can be diminished by adding a protection layer like JSR photoresist or eliminated using the metal mask on top of the polymer substrate during CO_2_ laser processing. It is important to keep hydrophilicity for capillary flow in microfluidics. The O_2_ plasma surface modification is used in common but a quick hydrophobic recovery is inevitable. The best modification of PDMS is obtained by combing the O_2_ plasma and PEG coating treatment which can retain the contact angle to be less than 70° for a long-term hydrophilic surface more than 420 h. This method is low-cost, disposable, and easy to integrate the microfluidic LOC system.

In the PDMS-based microfluidics, the PEG-coated PDMS meander capillary micromixer is a design example of the long-term hydrophilic chip using numerical simulation and verified by experiments for flow behavior and mixing performance. The simulated mixing extent of the meander mixing channel is in good agreement with experiments with a high mixing efficiency of 92%. In addition, an efficient passive planar rhombic micromixer with baffles in the microchannel is reviewed on the design simulation and experiment. The better fluid mixing is obtained at the smaller blockage ratio and higher Reynolds number due to the increased local average velocity and interfacial area. This rhombic micromixer with less mixing units and a short channel length has advantages of a good mixing efficiency at a low pressure drop for easy integration in a microsystem compared to conventional ones at a high pressure drop at a high Re of more than 100. Another design case of the simple planar obstacled micromixer with a short mixing distance is reviewed for effective mixing at low flow rates at a very low Re. From the numerical results of simulations and experimental confocal flow visualizations, the obstacled micromixer can achieve over 90% of mixing at *Re* <0.1 by molecular diffusion. The efficient micromixer is further integrated into the microreactor chip for silica nanoparticles synthesis. The synthetic particle size can be controlled by adjusting the reaction time and reaction temperature. It offers the possibility of high-throughput microfluidic synthesis for nanomaterial applications.

The emerging sustainable energy with PDMS triboelectric films of various microstructures for TENGs fabrication is reviewed in short, especially using the low-cost, easy-fabricated method of CO_2_ laser ablation and polymer casting for the case study in design and application. The high-surface-contact-area microneedles arrays with the separate and overlapped ones are designed for the Al/PDMS MN-TENG. Increasing the total effective contact area will enhance performance, particularly for the complex morphology of two-height overlapped microneedles TENG. The TENGs show a high output performance, sustainable electrical charging ability, and good storage energy ability. They are also applied to force or pressure sensors and various self-powered devices such as advertising boards, calculator, and temperature sensors. It evidences that the TENGs can promote harvesting wasted mechanical energy for a variety of practical power generation and an increasing number of sustainable energy applications in the near future.

## Figures and Tables

**Table 1:** Triboelectric tendency of materials: Triboelectric tendency series for common materials in TENG by easily losing electrons (positive) and/or getting electrons (negative).

Positive 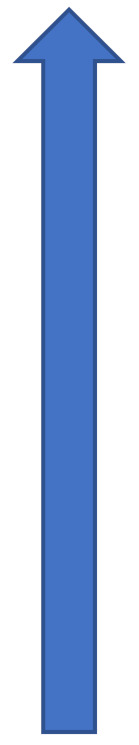	Polyamide 11	Polyvinyl alcohol (PVA)	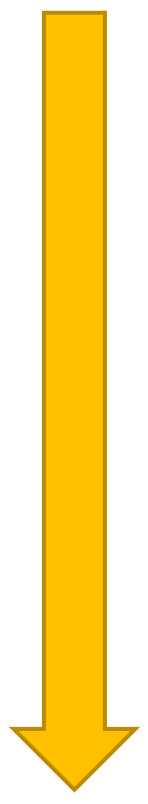 Negative
	Polyamide 6-6	Polyester	
	Melanimel formol	Polyuretane flexible sponge	
	Wool	Polyethylene Terephthalate	
	Silk	Polychlorobutadiene	
	Aluminum	Nature rubber	
	Paper	Polybisphenol carbonate	
	Cotton	Polychloroether	
	Steel	Polystyrene	
	Wood	Polyethylene	
	Hard rubber	Polypropylene	
	Nickel, Copper	Polyimide (Kapton)	
	Sulfur	Polyvinyl chloride (PVC)	
	Brass, Silver	Polydimethylsiloxane (PDMS)	
	Polymethyl methacrylate (Lucite)	Polytetrafluoroethylene (Teflon)	

**Table 2:** Comparison of surface morphology, materials, fabrication methods, operation condition, and electrical characteristics. [33].

Morphology	Materials	Fabrication Method	Operation Condition	Electrical Characteristics			Lighting (LEDs)	Ref
				Voc [V]	Isc [μA]	J [μA cm^−2^]		
Pyramid Line Cube	ITO/PET-PDMS/ PET/ITO	Photolithography, etching	Linear motor	18	0.7	0.13		[159]
				Pyramid > cube > line				
Nano pattern Textile	Ag/PDMS-ZnO/Ag	Chemical treatment, dip-coating	Mechanical force stimulator (10 kgf)	120	65	-		[162]
Nano-pillar Nano-Dome	Au/PDMS/Au	ICP etcher, thermal oxidation	130 N 3 Hz	83 60	3.2 2.5	-	75 -	[163]
Wrinkle (1D-40% PS-TENG)	Au deposited PS/ PTFE/Au	Thermal evaporation	65 N 3 Hz	83	14	3.5	160	[164]
Micropillars	Al/PDMS/Al	Soft lithography plasma treated	10 N 5 Hz	72	8.3			[167]
Microneedle	Al/PDMS/Al	CO2 laser ablation	Hand tapping, 3 Hz	102.8	43.1	1.5	53	[30]
OL-MN	Al/PDMS/Al	CO2 laser ablation	Hand tapping, 3 Hz	123	109.7	3.6	103	[32]
OL-TH-MN	Al/PDMS/Al	CO2 laser ablation	Hand tapping, 3 Hz	127	117.6	3.9		[33]
OL-DTH-MN	Al/PDMS/Al	CO2 laser ablation	Hand tapping, 3 Hz	167	129.3	4.3	226	[33]

**Table 3:** Nanoparticle synthesis: A recipe of the nanoparticle synthesis at a flow rate with *Re* 60.

		Alcohol (mL)	TEOS (g)	NH_4_OH (g)	DI Water (g)
Synthesis	Stream 1 (main inlet)	30	1.68		
at *Re* 60	Stream 2 (side inlets)	30		2	6

## Data Availability

Data are the author’s new Figure or prior publication in the references and the licences.
